# VHH CDR-H3 conformation is determined by VH germline usage

**DOI:** 10.1038/s42003-023-05241-y

**Published:** 2023-08-19

**Authors:** Zahra Bahrami Dizicheh, I-Ling Chen, Patrick Koenig

**Affiliations:** 23andMe, Inc. Therapeutics, 349 Oyster Point Boulevard, South San Francisco, CA 94080 USA

**Keywords:** Adaptive immunity, Protein folding, Structural biology

## Abstract

VHHs or nanobodies are single antigen binding domains originating from camelid heavy-chain antibodies. They are used as diagnostic and research tools and in a variety of therapeutic molecules. Analyzing variable domain structures from llama and alpaca we found that VHHs can be classified into two large structural clusters based on their CDR-H3 conformation. Extended CDR-H3 loops protrude into the solvent, whereas kinked CDR-H3 loops fold back onto framework regions. Both major families have distinct properties in terms of their CDR-H3 secondary structure, how their CDR-H3 interacts with the framework region and how they bind to antigens. We show that the CDR-H3 conformation of VHHs correlates with the germline from which the antibodies are derived: IGHV3-3 derived antibodies almost exclusively adopt a kinked CDR-H3 conformation while the CDR-H3 adopts an extended structure in most IGHV3S53 derived antibodies. We do not observe any bias stemming from V(D)J recombination in llama immune repertoires, suggesting that the correlation is the result of selection processes during B-cell development. Our findings demonstrate a previously undescribed impact of germline usage on antigen interaction and contribute to a better understanding on how properties of the antibody framework shape the immune repertoire.

## Abstract (web summary)

Analysis of existing structures of VHHs (nanobodies) in combination with deep sequencing of the immune repertoire of Llamas provides evidence for the impact of VH gene usage on the VHH CDR-H3 conformation.

## Introduction

Heavy-chain antibodies are a unique type of antibodies found in camelids (camels, llamas, and alpacas). The antigen binding region (paratope) of heavy-chain antibodies is solely formed by a single variable domain called VHH or nanobody^1^ (Supplementary Fig. 1). The small size and in some cases, stable fold of VHH domains, have led to the use of VHH domains in diagnostic and imaging studies^2^. Moreover, they serve as crucial building blocks in a variety of pre-clinically and clinically investigated multivalent and multi-specific antibodies in therapeutic areas like oncology^3,4^ and infectious diseases^5^.

Most camelid heavy-chain antibodies are derived from dedicated IGHV genes. While those genes show high sequence identity to the human VH3 family, they carry differences in comparison to variable domains of conventional antibodies. Those differences are thought to allow them to act in the absence of a paired light chain. Among these differences are a longer complementarity determining region H3 (CDR-H3) loops^6–8^, substitutions in the framework 2 (FWR2) region, which is responsible for heavy–light chain pairing in conventional heavy-chain (HC)/light chain (LC) antibodies^1,9,10^ and non-canonical disulfide bonds^8,11^. The FWR2 substitutions, also referred to as ‘hallmark residues’, consist of changes from hydrophobic to more hydrophilic residues, which are believed to lead to a more soluble interface in the absence of the light chain. However, hallmark residues are not a requirement for the heavy-chain antibody ontogeny, as antibodies from the IGHV3 and IGHV4 germline families in which those hallmark residues are absent are also able to form heavy-chain antibodies in camelids^12,13^. Another feature of some heavy-chain-antibodies is an additional non-canonical disulfide bond between CDR-H1 or CDR-H2 and CDR-H3^8,11^. The Cys in CDR-H1 and CDR-H2 are germline-encoded, which suggests an evolutionary functional role for the covalent bond^11^. The additional disulfide bond might reduce the entropic penalty associated with long CDR-H3s^14^ and lead to great thermal stability in some antibodies^15^. Overall, the heavy-chain antibody immune repertoire is dominated by antibodies derived from a handful of germlines. While alpacas carry 88 IGHV genes^12^, sequencing of the VHH repertoire from llama and alpaca revealed that the VHH repertoire in those animals is formed by antibodies that are derived from very few IGHV germlines and carry hallmark residues; these germlines include IGHV3-3, IGHV3S53, and a germline which carries a non-canonical cysteine in CDR-H2 (often IGHV3S61, IGHV3S65, or IGHV3S66)^6,7^.

Despite the sequence similarity of many VHH germlines to the human VH3 family, the mode of antigen binding and CDR loop structures of VHH domains differs significantly from conventional heterodimeric antibodies. CDR1 and CDR2 of VHHs often do not cluster into previously identified canonical heavy-chain CDR conformations^16,17^. Long CDR-H3 loops in many VHH domains adopt a stretch-twist-turn or kinked conformation where the CDR-H3 loops cover the interface that interacts with the light chain in conventional HC/LC antibodies^18,19^. A shared feature of this kinked CDR-H3 conformation is a hydrophobic core formed by several aromatic residues from CDR-H3 and FWR2^18–20^. In contrast other VHH antibodies contain a CDR-H3 which adopts an extended conformation (e.g., refs. ^21–23^), which is uncommon in human antibodies^24^. As a result of the monomeric nature as well as different CDR loop conformations, antigen binding of VHH domains is dominated by the CDR-H3 loop. For example, in the case of anti-lysozyme antibodies more than 50% of the paratope area is provided by the CDR-H3 loop^17^. In addition, VHH domains often utilize the antibody framework region for antigen binding^25^. While there are examples of a planar paratope in VHH antibodies (for example refs. ^22,26^), VHH antibodies are also able to form a unique convex paratope involving the CDR1 or CDR2 and the extended conformation CDR-H3 allowing VHHs to recognize unique epitopes like enzymatic pockets which are not commonly recognized by heterodimeric antibodies^17^.

Past research has studied the structural properties of VHH CDR loops in sets of VHH antibody structures contributing to an understanding of VHH CDR loop conformation and antigen interaction^16,18,19,25^. However, our understanding of the overall heterogeneity of different CDR-H3 loop conformations in VHH domains, their functional consequences, as well as the origin of the CDR-H3 heterogeneity, is limited. To address these questions, we combined a large dataset of publicly available structural data with immune repertoire sequencing data to identify factors that shape the CDR-H3 conformation in VHH domains.

## Results

### Most VHH antibodies carry either an extended or a kinked CDR-H3 loop

**Fig. 1:**
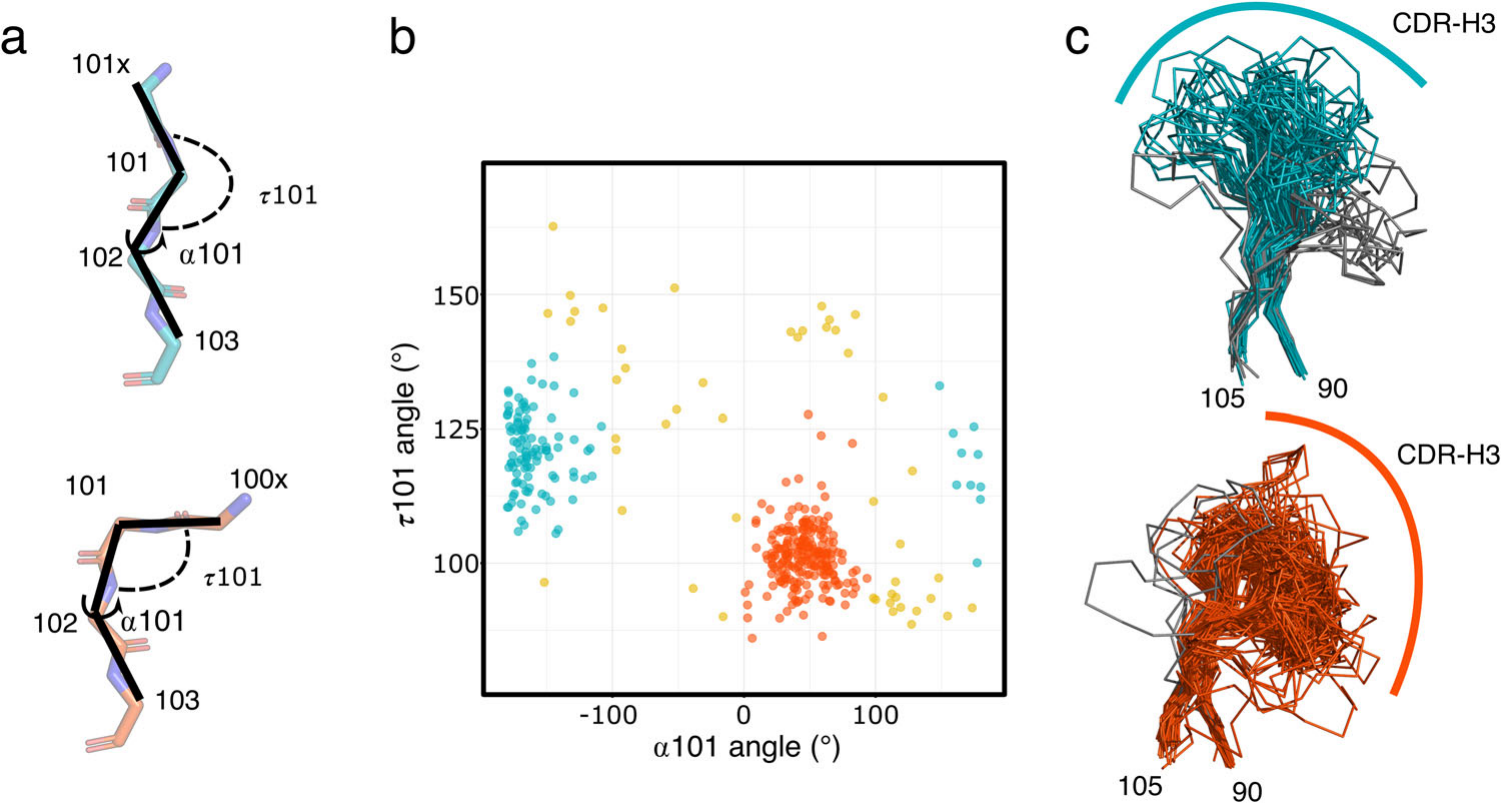
Clustering of the CDR-H3 loop of VHH antibodies based on the conformation of the C-terminal stem of the loop. **a** Representative conformation of the C-terminal stem of CDR-H3 for extended (top, teal) and kinked (bottom, red) structures are shown. The angles α101 and τ101 which are used for CDR-H3 loop classification are highlighted. **b** α101 and τ101 angle distributions in a unique set of VHH structures from llama and alpaca (*n* = 385 VHH structures). Structures highlighted in blue were classified as having an extended CDR-H3 conformation and structures highlighted in red a kinked CDR-H3 conformation. **c** Aligned CDR-H3 regions which were clustered as extended (top, teal) and as kinked (bottom, red). Structures that were removed from the subsequent analysis are colored in gray (see main text for details).

To systematically interrogate the structural conformations of CDR-H3 loops in VHH domains, we aggregated a dataset of 385 unique VHH structures from the llama genus (*llama glama, llama vicugna, and llama pacos*) deposited in the Protein Data Bank (PDB). The CDR-H3 loop in antibodies is structurally the most diverse of all CDR loops. In contrast to other CDR loops, CDR-H3 loops cannot be clustered into canonical loop conformations. However, the H3 loop in conventional HC/LC antibodies has been previously classified into two groups based on the conformation at the C-terminus of H3 involving positions 100x, 101, and 102: loops with an extended and a kinked C-terminal CDR-H3 (Chothia numbering scheme is used throughout the manuscript unless otherwise noted, Fig. 1a)^24,27^. We applied the previously established metric for HC/LC antibody CDR-H3 loop classification to analyze the conformational heterogeneity of CDR-H3 loops in VHH structures. To capture the C-terminal H3 conformation we measured two main chain backbone angles: (1) τ101 angle which is a pseudo dihedral angle of the Cɑ atoms of 100x, 101, and 102 and (2) the ɑ101 angle^24^ (Fig. 1a). Based on the τ101 and ɑ101 angles, structures can be separated in two large clusters: 58% of structures follow a kinked geometry in their CDR-H3 (0 < ɑ101 < 120°, 85° < τ101 < 130°) and 29% of structures follow an extended CDR-H3 geometry (−100° > ɑ101, 100° < τ101 < 145°) (Fig. 1b). The VHH distribution of structures with kinked and extended CDR-H3 loop geometry deviates from human structures where 79% of antibodies have a kinked CDR-H3^24^.

In contrast to conventional HC/LC antibodies, the CDR-H3 loop of VHH domains can adopt a larger conformation space as it is not restricted by the paired light chain domain. In VHH antibodies, the CDR-H3 loops often fold back onto the part of the VHH structure which in conventional HC/LC antibodies would interact with the light chain. The former light chain interaction interface is made up of framework (FWR) 2 and part of FWR3 of the variable domain. A structural alignment of the CDR-H3 loop regions reveals that most structures with a kinked CDR-H3 fold back onto FWR2 and FWR3 region (Fig. 1c). In contrast, in VHH domain structures with an extended CDR-H3 loop conformation the CDR-H3 loop interacts less with FWR2 and FWR3 (Fig. 1c).

A few structures, which were classified as having an extended or kinked CDR-H3 conformation, appear to deviate from the assigned conformation. For example, some CDR-H3 loops classified as extended appear nevertheless to interact with framework 2 as their CDR-H3 kinks are not at position 101 but at a different position leading to the miss-classification of those antibodies. We manually removed those outlier structures from further analysis (Fig. 1c and Supplementary Fig. 10).

We also measured the length of all three CDRs in the two structural CDR-H3 clusters. While we did not find any significant CDR-H1 length differences (mean length of 7 amino acids for both kinked and extended structures, Supplementary Fig. 2a), the CDR-H2 loop lengths differ by about one amino acid (mean length of 6 and 5 amino acids for kinked and extended structures, Supplementary Fig. 2b). Furthermore, CDR-H3 loops are significantly longer in kinked structures when compared to the extended structures (12 vs. 9 amino acids, *p*-value 3.6e-20, Supplementary Fig. 2c). The length of the CDR-H3 loops likely reflects different structural requirements to adapt the different CDR-H3 conformations.

### VHH structures with kinked and extended CDR-H3 show distinct differences in their FWR2 and CDR-H3 primary sequence composition

**Fig. 2:**
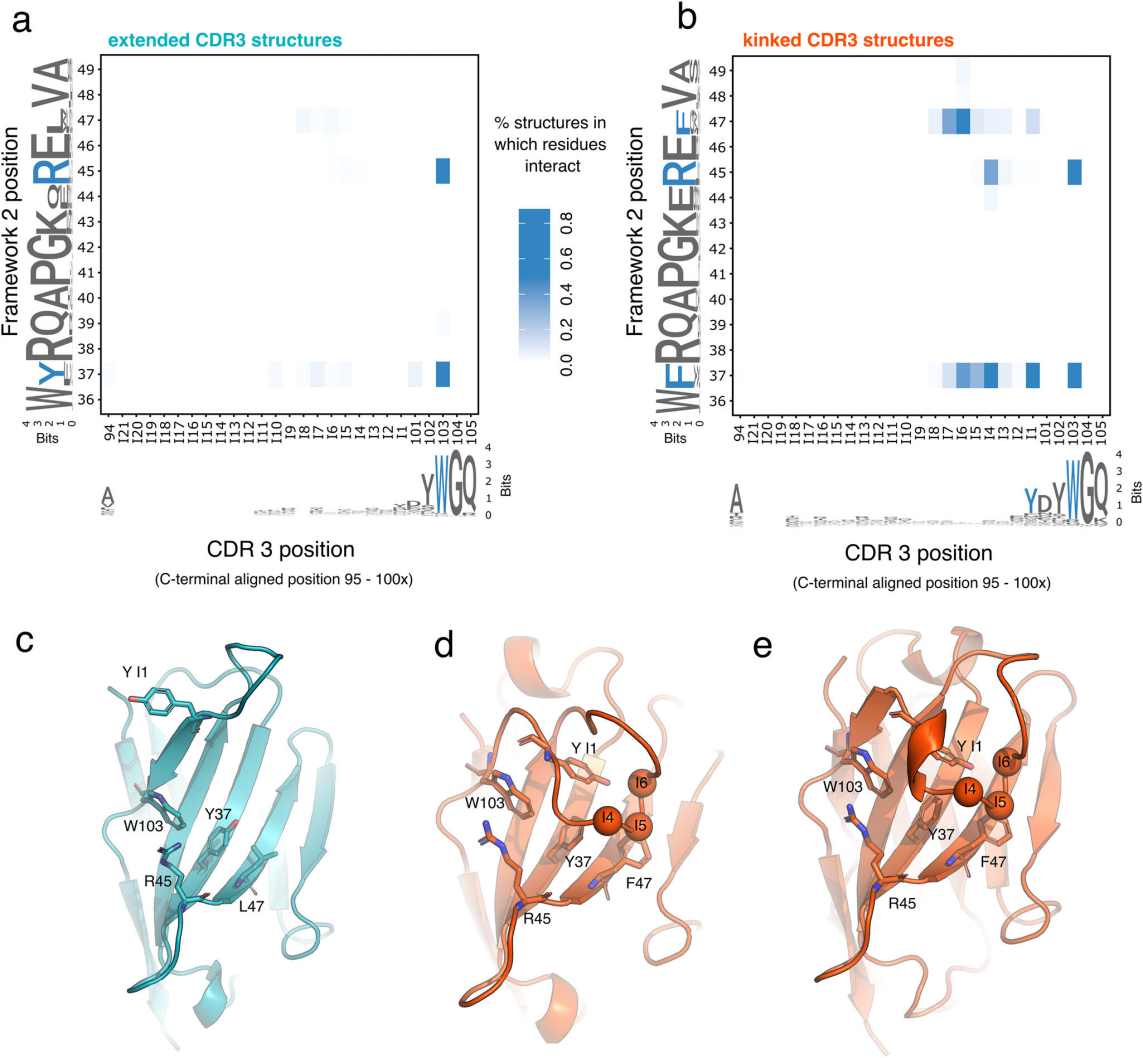
Sequence and structural differences in the CDR-H3 and framework of the two structural CDR-H3 clusters. **a** Contact map between CDR-H3 and FWR2 in extended CDR-H3 structures (*n* = 100). The blue heatmap intensity represents the fraction of structures where a particular pair of residues interact with each other (see main text for details). FWR2 and CDR-H3 positions were numbered using the Chothia numbering scheme, except for CDR positions 95 to 100x which were aligned from the C-terminus to reveal conserved interactions at the C-terminus of CDR-H3. The logo plots at the X and Y-axis of the heatmap were generated from the sequences used in the contact analysis. **b** Contact map as in *a* for structures carrying kinked CDR-H3 conformation (*n* = 215). **c** Cartoon representation of a typical extended CDR-H3 VHH structure (PDB code 4LDE.A) with key residues in CDR-H3 and FWR shown in stick representation. **d** Cartoon representation of a typical kinked CDR-H3 VHH structure (PDB code 3K7U.A) with key residues in CDR-H3 and FWR shown as in stick representation. **e** Cartoon representation of a typical kinked CDR-H3 VHH structure (PDB code 1U0Q.A) carrying a short 3–10 helix in CDR-H3; key residues in CDR-H3 and FWR are shown in stick representation.

To better understand which amino acid positions are crucial for the two different conformations in the two CDR-H3 clusters, we generated a contact map between FWR2 and the CDR-H3 loops (Fig. 2a, b). Residues that were distanced 4 Å or less apart from each other were classified as being in contact. Since we have demonstrated a correlation between the conformation of the C-terminal region of CDR-H3 (position 100x, 101, and 102) and the overall conformation of the CDR-H3 loop, the CDR-H3 loop was C-terminally aligned for the analysis to identified potential conserved elements at the C-terminus of H3 which might interact with FWR2. Residues were numbered backward starting from position 101X (named in the analysis insert position 1 (I1)) to position 95 (I21).

The distance analysis confirms that most of the structures which have an extended CDR-H3 loop do not interact with FWR2 except for a conserved pi-stacking interaction between CDR-H3 position 103 and FWR2 positions 37 and 45 (Fig. 2a, c). Kinked CDR-H3 structures however share multiple interactions between CDR-H3 and FWR2 (Fig. 2b). These interactions involve distinct residues at FWR2 (pos. 37, 45, and pos 47) and CDR-H3 (pos 101 and 103 as well as position I1, I4 and I6 of the C-terminally aligned CDR-H3 loop and can be grouped into clusters. The first one consists of pie-stacking interactions of residues Arg45, Trp103, and Tyr at I1. The second cluster consists of the backbone interaction of positions I4, I5, and I6 with the mostly large aromatic side chains of pos 37 and 47 (Fig. 2e).

Next, we ask if the two CDR-H3 loop conformational clusters show differences in the amino acid sequences of CDR-H3 or FWR2. A sequence logo analysis demonstrates that the C-terminal positions of both CDR-H3 clusters share the consensus sequence YDYWGQ (pos I1 to pos 105). The major difference appears to be the lower conservation of Tyr and the Asp at pos I1 and 101 in extended structures (Fig. 2a, b). The amino acid sequences of FWR2 show distinct differences between the conformational clusters. While kinked structures carry a Phe at positions 37 and 47, extended CDR-H3 structures carry a Tyr and a Leu at those positions. In addition, while position 44 is mostly a Gln in VHH structures with kinked CDR-H3 loops, the position is less conserved in extended CDR-H3 structures. Furthermore, we identified differences in the secondary structure of CDR-H3 between the two clusters. We find that kinked CDR-H3 loops share a common motif at positions I2 to I4 where 75% carry either a short 3-10 helix or a turn motif at those residues (Supplementary Fig. 3a, b), while the secondary structure of extended CDR-H3 loops is dominated by a strand-turn-strand motif. This observation is in line with a previous report demonstrating that longer CDR-H3 loops in VHH contain helical structure elements^18^.

### VHH kinked and extended CDR-H3 structures interact differently with antigen

**Fig. 3:**
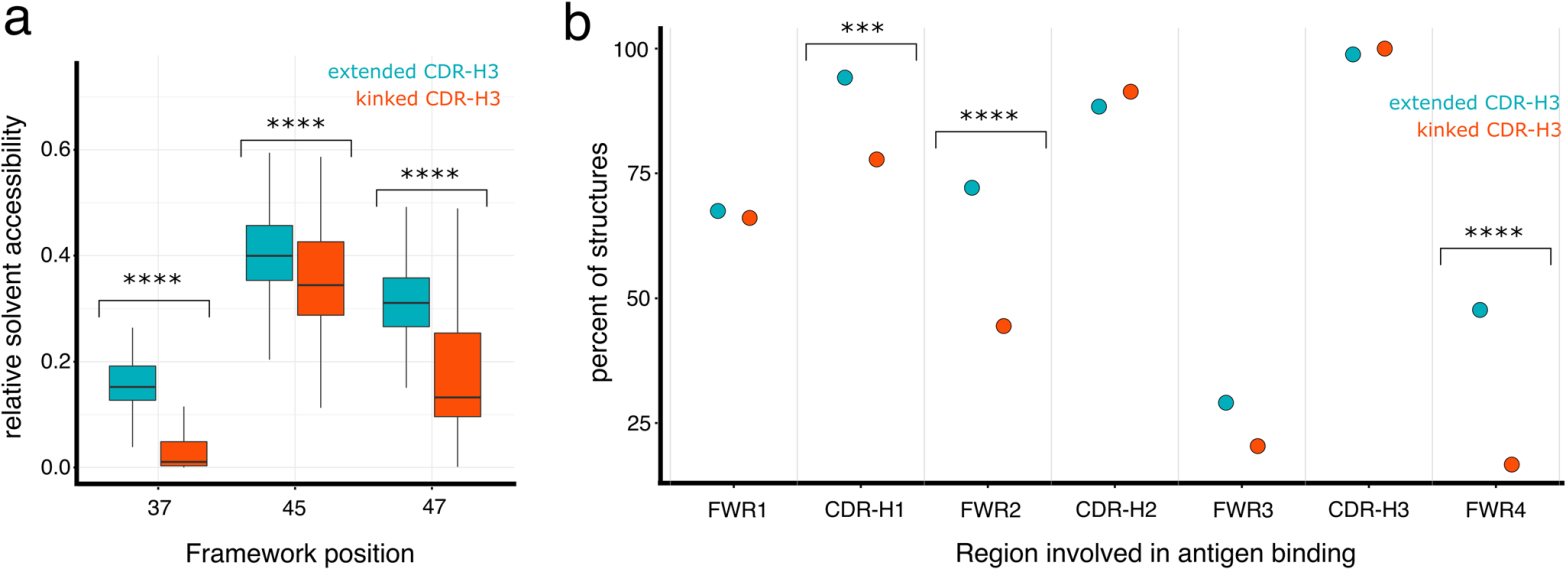
The solvent-exposed framework 2 region of antibodies with an extended CDR-H3 loop is frequently involved in antigen binding. **a** Boxplot of the relative solvent accessible area of FWR2 position 37, 45, and 47 in VHH structures with extended (teal, *n* = 100) or kinked CDR-H3 (red, *n* = 215) (Wilcoxon rank test adjusted *p*-values, pos 37: 7.7e-32, pos 45: 1.7e-5, pos47: 1.7e-21). **b** Percent of VHH structures with a kinked (red, *n* = 162) and extended (teal, *n* = 86) CDR-H3 which use the different regions (FWR1-4, CDR-H1-3) of the VHH domain for interaction with their respective antigen. Regions that show a significant difference based on a Chi-squared test are labeled (*p*-values CDR1: 1.7e-3, FWR2: 5.7e-5, FWR4: 4.2e-7).

Given the large structural differences evident in kinked and extended CDR-H3 loops, we investigated if different CDR-H3 conformation results in differences in antigen binding. In the absence of the light chain, VHH domains often use the antibody framework to increase the antigen interaction area^25^. As the two structural clusters differ in how the CDR-H3 interacts with the FWR2, we first measured the relative solvent accessibility area (RSA) of positions 37, 45, and 47. As expected, due to the minimal interaction between FWR2 and CDR-H3 in extended CDR-H3 structures, FWR2 positions 37 and 47 in extended CDR-H3 are more solvent exposed in extended than in kinked CDR-H3 structures (Fig. 3a).

Next, we ask if the difference in FWR2 solvent exposure and the different CDR-H3 loop conformations have any impact on how the antibodies interact with the antigen. We identified 279 unique protein antigen-bound VHH structures including 162 with a kinked and 86 with an extended CDR-H3 conformation. We grouped the structures based on their mode of antigen binding (Fig. 3b) and analyzed which region of the VHH (FWR1, CDR-H1, FWR2, CDR-H2, FWR3, and CDR-H3) is in contact with the antigen. In more than 72% of all extended structures, FWR2 is part of the paratope while this is the case for only more than 44% of kinked CDR-H3 structures. A common paratope of extended CDR-H3 structures consists of CDR-H1, CDR-H2, CDR-H3, and FWR2 (about 30% of all paratopes) while this type of paratope is only present in 10% of kinked structures (Supplementary Fig. 4). A similar picture emerges when we look at the area each VHH region contributes to the paratope (Table 1). The mean paratope area contributed by FWR2 is larger in antibodies with extended CDR-H3 structures than in antibodies with kinked CDR-H3 structures (82Å^2^ vs 32Å^2^), while the paratope contribution of CDR-H3 for both structural clusters is very similar (kinked CDR-H3 411 Å^2^, extended CDR-H3 structures 400 Å^2^).

**Table 1:** Mean paratope contributions from different regions of the VHH domain in VHH structures containing either a kinked or extended CDR-H3 conformation.

	FWR1 [Å^2^]	CDR1 (Å^2^)	FWR2 (Å^2^)	CDR2 (Å^2^)	FWR3 (Å^2^)	CDR-H3 (Å^2^)
Kinked CDR-H3 VHH (*n* = 170)	62.1 ± 87.8	49.2 ± 55.5	31.9 ± 67.5	174.5 ± 131.8	20 ± 56.4	411.3 ± 184.9
Extended CDR-H3 VHH (*n* = 92)	78 ± 99.2	64 ± 45.9	81.7 ± 87.1	170.7 ± 115.8	15.9 ± 46.5	400 ± 189
Adjusted *p*-value (significance)	1 (n.s.)	1.3e-02 (***)	2.7e-07 (****)	1 (n.s.)	1 (n.s.)	1 (n.s.)

Mean paratope area in Å^2^ ± standard deviation for variable domain regions as defined in the Chothia numbering scheme. Significance testing is performed using a Wilcoxon rank test. n.s.: non-significant, ***<=0.001, ****<=0.0001.

In summary, we demonstrate that CDR-H3 loops of VHH structures can be classified based on their conformation at position 100x-102 into two structural clusters. The first cluster contains kinked CDR-H3 conformations with longer CDR-H3 loops, which mostly fold back on FWR2 exhibiting distinct conserved interaction between FWR2 and CDR-H3. The second cluster has an extended loop conformation with a shorter average CDR-H3 loop length where the CDR-H3s mostly protrude into the solvent and show no interaction with FWR2. The clusters further differ in their FWR2 and the CDR-H3 primary sequence. As a result of the different CDR-H3 loop conformations, VHH domains with extended CDR-H3 loops utilize the FWR2 more frequently for antigen binding.

### The VH germline correlates with CDR-H3 loop conformation

**Fig. 4:**
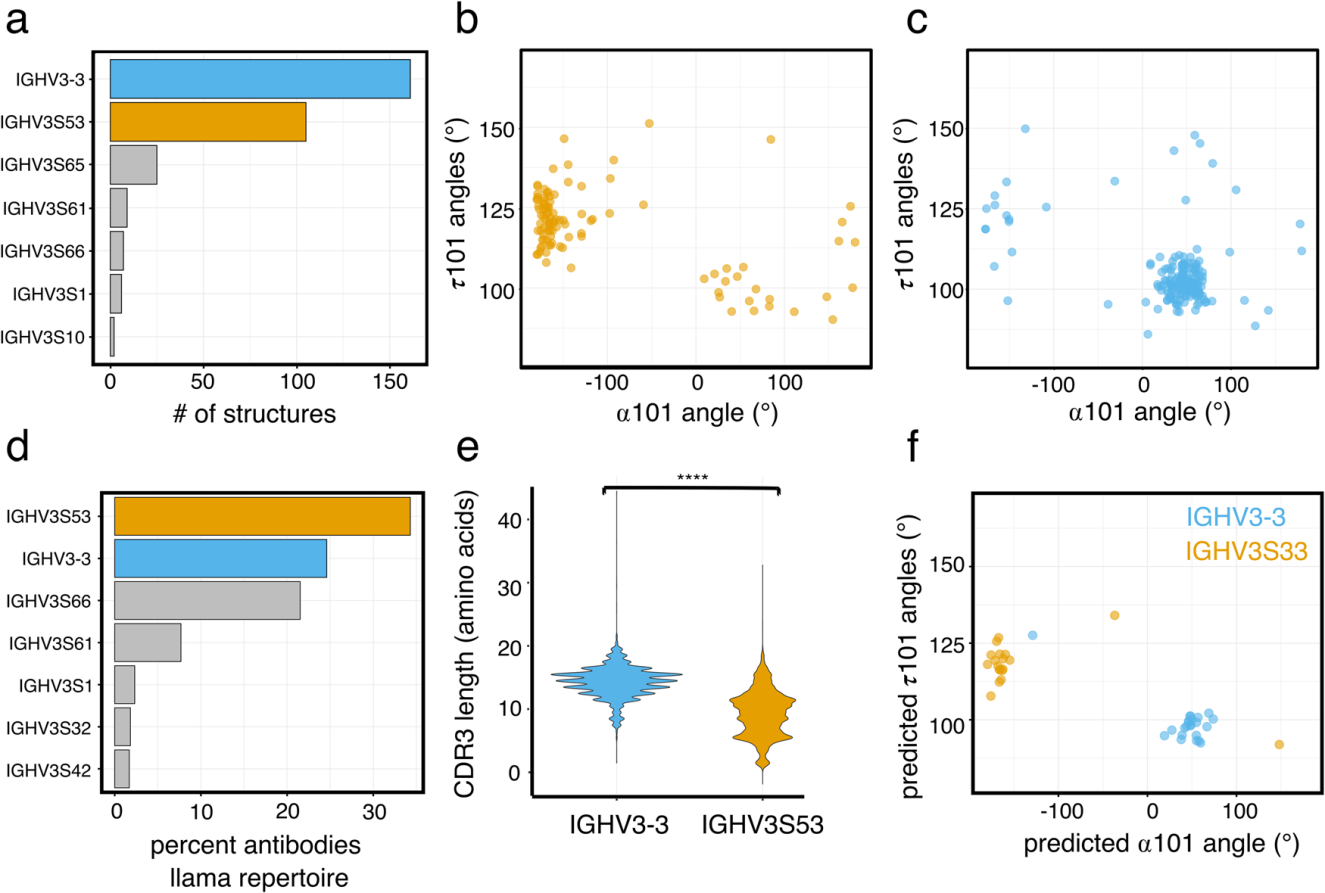
The two CDR-H3-based structural clusters differ in their HV germline usage. **a** Top 7 most frequent VH germline usage in the dataset of unique VHH structures (*n* = 385) used in this study. **b** α101 and τ101 angle distributions in a unique set of VHH structures (*n* = 106) derived from the IGHV3S53 germline. **c** α101 and τ101 angle distributions in a unique set of VHH structures (*n* = 175) derived from the IGHV3-3 germline. **d** Top 7 most frequent VH germlines used in the combined repertoire dataset of the two sequenced llama samples. **e** CDR-H3 amino acid length distribution in the combined repertoire of llama 1 and 2 for antibodies either derived from IGHV3-3 (*n* = 13462 sequences, blue) or IGHV3S53 (*n* = 18769 sequences, orange) germlines (Wilcoxon rank test adjusted *p*-value 2e-16). **f** α101 and τ101 angles predicted by Alphafold for 23 VHH sequences derived from the IGHV3-3 germline (blue) and 19 VHH sequences derived from the IGHV3S53 germline (orange).

We have identified differences in the sequence of framework 2 in extended and kinked structures at FWR2 positions 37, 45, and 47 (Fig. 2a, b). One explanation for those differences could be biases in VH germline usage. Alpaca VH germlines show relatively high sequence similarity but often differ at key positions in FWR2^12^. We, therefore, assigned alpaca VH germlines to the structures in our dataset. The analysis reveals that most structures originate from IGHV3S53 and IGHV3-3 germlines (Fig. 4a). However, the germline distribution is skewed when we look at the extended and kinked structures separately. 75% of all IGHV3S53-derived VHH structures adopt an extended CDR-H3 structure conformation, 82% of all IGHV3-3-derived VHH structures adopted a kinked CDR-H3 conformation (Fig. 4b, c). IGHV3-3 and IGHV3S53 differ in their FWR2 sequence. IGHV3-3 encodes for Phe, Glu, and Phe at positions 37, 45, and 47, respectively, while IGHV3S53 carries Try, Gln, and Leu at the same positions (Supplementary Fig. 5a). The difference in the FWR2 residues aligns with our previous observation for framework sequence differences (Fig. 2a, b). Further, the previously observed differences in CDR-H2 length fit the germline dependency of the CDR-H3 conformation. The IGHV3S53 germline carries a CDR-H2 of 7 amino acid length, while the CDR-H2 encoded by IGHV3-3 has a length of 8 amino acids (Supplementary Figs. 2b and 5b).

To independently confirm our observation of a correlation between germline usage and CDR-H3 loop conformation we turned to immune repertoire sequencing. Using a sequencing workflow designed to minimize primer amplification biases and sequencing errors (see details in Material and Methods), we obtained 21814 and 34915 BCR (B-cell receptor) sequences, respectively, from two llamas (Table 2). The immune repertoires are dominated by antibodies derived from the IGHV3-3, IGHV3S53, IGHV3S61, and IGHV3S66 germlines (Fig. 4d and Supplementary Fig. 6a, b). The VH germline distribution in the immune repertoire reflects the overall usage of germlines in our structure dataset (Fig. 4a). However, one exception is that IGHV3S61, IGHV3S65, and IGHV3S66 germlines are underrepresented in the structure dataset, likely because they contain a germline-encoded cysteine and thus are potentially less preferred in structural studies (Supplementary Fig. 7a, b). Analyzing CDR-H3, we further found that IGHV3-3-derived antibodies have a significantly longer CDR-H3 loop (mean length 14 amino acids) than IGHV3S53-derived antibodies (mean length 9 amino acids, Wilcoxon rank test adjusted *p*-value 2e-16) (Fig. 4e).

**Table 2:** Repertoire sequencing statistics.

Animal	Raw reads	Sequence reads with exactly 1 complete UMI	Unique UMIs with a frequency ≥10	Sequences with the complete in-frame variable domain
Llama1	1467432	1366353	24705	21129
Llama2	1519339	1388958	38032	34277

Finally, we used Alphafold^28^ to model the structures of VHH sequences obtained through repertoire sequencing. We first validated that Alphafold can correctly predict the CDR-H3 loop conformation. Using 35 kinked and 36 experimentally determined VHH structures which were selected randomly, we demonstrate that when modeling those structures with Alphafold, the program predicts the CDR-H3 loop conformation with high confidence (false positive and false negative rates for kinked structures 9% and 6%, respectively, and 6% and 8% for extended structures, respectively) (Supplementary Fig. 8a–i). We next used Alphafold to predict the structure of 20 IGHV3-3 derived and 20 IGHV3S53-derived antibody sequences, which were randomly chosen from the llama immune repertoires. 90% of IGHV3S53 structures adopt an extended CDR-H3 loop while more >95% of predicted IGHV3-3 adopt a kinked CDR-H3 loop (Fig. 4f).

In summary, we identify similar differences in the CDR-H3 properties of IGHV3-3 and IGHV3S53-derived antibodies in our repertoire sequencing dataset as we have previously shown using structural data supporting the idea that the structural dataset investigated is a representative sample of multiple immune repertoires.

### There is no strong bias in VDJ combination in the immune repertoire of llamas which could explain the VH germline-dependent clustering of CDR-H3 conformations

**Fig. 5:**
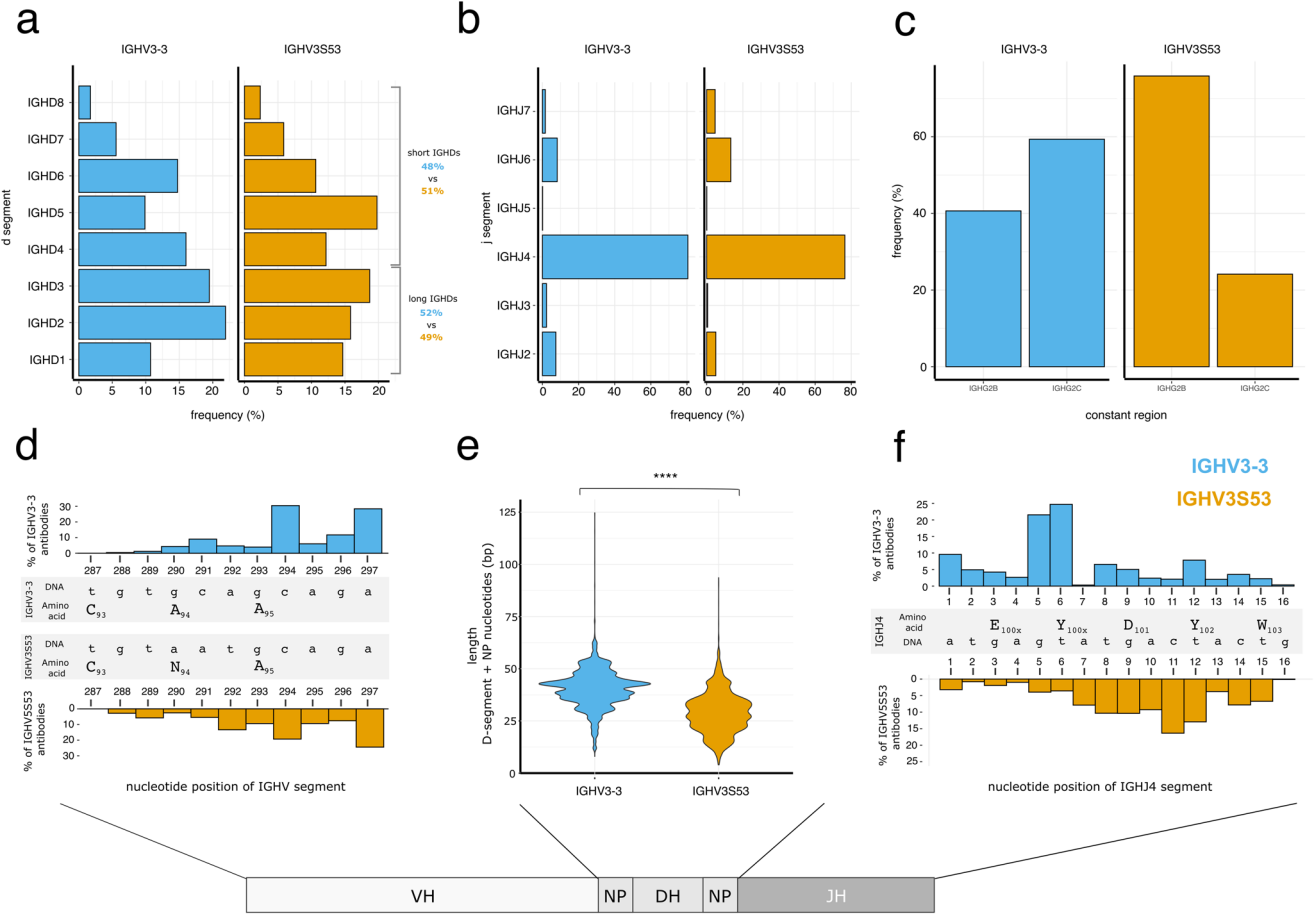
VDJ junction properties of IGHV3-3 and IGHV3S53-derived VHH antibodies. **a** IGHD segment usage in the combined repertoire of llama 1 and 2 for antibodies either derived from IGHV3-3 (blue) or IGHV3S53 (orange) germlines. **b** IGHJ segment usage in the combined repertoire of llama 1 and 2 for antibodies either derived from IGHV3-3 (blue) or IGHV3S53 (orange) germlines. **c** Llama IgG2 distribution in the combined repertoire of llama 1 and 2 for antibodies either derived from IGHV3-3 (blue) or IGHV3S53 (orange) germlines. **d** The histogram depicts the distribution of the most 3’ prime nucleotide position in the VH segment of antibodies derived from an IGHV3-3 (upper panel) and IGHV3S53 germline (lower panel). **e** Nucleotide length distribution of NP nucleotides and D-segment of antibodies either derived from IGHV3-3 (*n* = 10304 sequences, blue) or IGHV3S53 (*n* = 14451 sequences, orange) VH germlines (Wilcoxon rank test adjusted *p*-value 2e-16). **f** The histogram depicts the distribution of the most 5’ prime nucleotide position in the IGHJ4 segment of antibodies derived from IGHV3-3 (upper panel) and IGHV3S53 germlines (lower panel).

The CDR-H3 loop is encoded by the combination of the VH, DH, and JH segments. To answer the question if biases also exist in DH and JH usage for the two different CDR-H3 structural clusters, we turned to our llama immune repertoire sequencing datasets. We first compared the DH and JH usage between IGHV3-3 and IGHV3S53-derived antibodies (Fig. 5a). DH genes can be classified into two different groups: long D_H_ segments (IGHD1 to IGHD3) which are between 29-34 nucleotides long and short DH segments (IGHD4 to IGHD8) which have length of 16–19 nucleotides (Supplementary Fig. 9). The distribution of those DH segments is nearly identical for IGHV3-3 and IGHV3S53-derived antibodies: The percent of IGHV3-3 and IGHV3S53 segments which pair with long DH segments is 52% and 49%, respectively. The small difference unlikely accounts for the differences in CDR-H3 loop length observed. We also investigated the JH-segment pairing for IGHV3-3 and IGHV3S53 and found that the JH-segment usage is similar between the two different VH-gene-derived antibodies. The JH usage is dominated by IGHJ4 (Fig. 5b).

Our sequencing strategy allowed us also to assign the constant region IGH2B and IGH2C isotypes that are associated with domain antibodies to the obtained variable domain sequences. We do observe differences in constant isotype usage between IGHV3-3 and IGHV3S53. While more than 70% of all IGHV3S53 have an IGH2B isotype, more than 60% of all IGHV3-3 sequences have a constant region which is IGH2C (Fig. 5c). However, given that we also see large variations between the two animals (Supplementary Fig. 6c, d), it is unclear if this is related to VH germline usage or more likely a result of animal-specific variations. In summary, the differences in CDR-H3 loop conformations cannot be explained by biases in the VDJ recombination as the DH and JH germline usage is nearly identical for IGHV3-3 and IGHV3S53-derived domain antibodies.

VH_,_ DH, and JH gene usage is not the only factor impacting CDR-H3 length. During VDJ recombination the gene segments are trimmed by nuclease activity and the random addition of N-nucleotides has an impact on CDR-H3 length. To understand if antibodies are trimmed differently, we investigated the trimming of VH and JH segments in antibodies originating from IGHV3-3/IGHJ4 and IGHV3S53/IGHJ4 germlines. We choose those VH/JH combinations as most IGHV3-3 and IGHV3S53 antibodies carry a JH4-derived J-segment. We find that both VH segments are trimmed at similar positions, mainly base pair 294 and 297, which preserves the structurally important Cys93 (Fig. 5d). Next, we analyzed DH segment processing in IGHV3S53 and IGHV3-3 based antibodies (Fig. 5e). We determined the length of the combined DH and NP-nucleotide segment. The results show that the DH/NP segment length mirrors the overall CDR-H3 length difference between IGHV3S53 and IGHV3-3-based VHHs. Finally, both antibody groups differ in how their IGHJ4 segment is trimmed. IGHV3-3 based antibodies are trimmed at base pair 5 and 6, which retains Tyr101x (Fig. 5f). In the majority of IGHV3S53 base antibodies JH4 trimming occurs at base pair 7 to 12, preserving Tyr102 but not Tyr101x. The difference in JH trimming is evident in the CDR3 sequence conservation where Tyr101x is less conserved in IGHV3S53 antibodies (Fig. 2a, b, sequence logos). In summary, the CDR-H3 loop length difference between IGHV3S53 and IGHV3-3-based antibodies stems from differences in JH trimming and in DH/NP segment lengths. We observe no difference in the trimming of the VH segment.

### Composition of framework 2 impacts the CDR-H3 conformation of kinked CDR-H3 loops

**Fig. 6:**
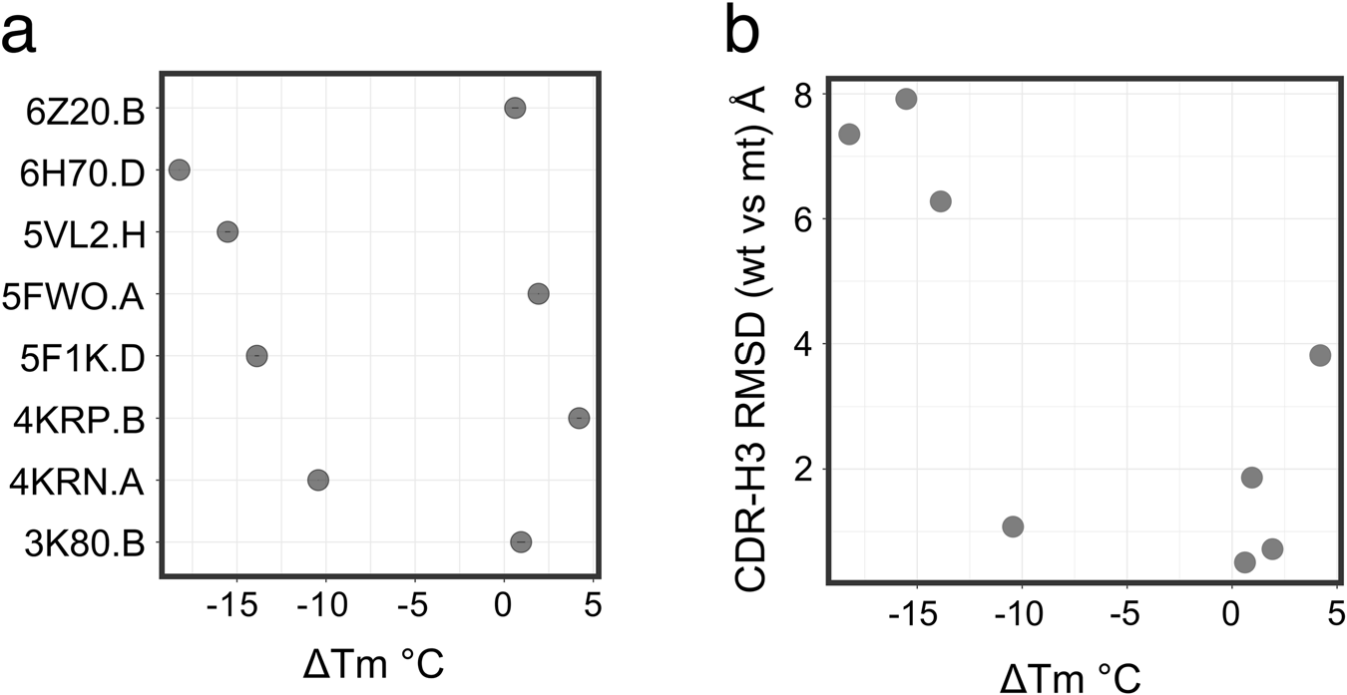
Framework 2 residue composition at positions 37 and 47 impact stability and CDR-H3 conformation. **a** Changes in melting temperature (Δ*T*_M_) between the wildtype version of selected VHHs and their respective F37Y/F47Y mutant version. PDB code and respective chains are listed on the Y-axis. The standard deviation of three technical replicates is represented using an error bar. **b** Scatter plot between change in melting temperature (Δ*T*_M_) and change in CDR-H3 conformation (root-mean-square deviation; RMSD) as predicted by Omegafold for the VHH molecules shown in *a*.

Since we do not find any indication that the correlation between VH germline usage and CDR-H3 conformation is the result of biases in VDJ processing and recombination, we ask whether the framework directly impacts the CDR-H3 conformation. A recent structural study demonstrated that when a VHH, called 7D12, which is derived from the IGHV3-3 germline and which adopts a kinked CDR-H3 conformation is grafted onto a humanized framework that carries a IGHV3S53-like FWR2, the previously kinked CDR-H3 adopts an extended conformation which leads to a drastic reduction in antibody thermostability^29^. To understand if the change in thermostability and CDR-H3 conformation is a specific behavior of 7D12 or if the impact of FWR2 on CDR-H3 conformation is a more general feature in VHHs, we selected 8 VHH antibodies from the PDB, which have a kinked CDR-H3 structure and are derived from IGHV3-3 germline, for expression and purification. We further generated for each VHH a variant that contained two mutations in FWR2, F37Y, and F47L, resulting in an IGHV3S53-like FWR2. As in the previous study^29^, we measured the melting temperature (*T*_m_) of the wildtype VHHs and compared it to the respective FWR2 variants (Δ*T*_m_) (Fig. 6a). Half of the VHHs tested showed a large negative Δ*T*_m_ of −10 °C demonstrating that they get destabilized by the introduction of the framework mutations. Next, we used the structural prediction program Omegafold^30^ to predict the impact of the mutations on the VHH structure (Fig. 6b). Omegafold has been shown to predict the overall VHH CDR-H3 conformation among different machine learning-based structure prediction tools with the highest accuracy^31^. Omegafold predicted for three of the four VHHs which show a decrease in *T*_m_ a large change in CDR-H3 conformation as measured by the root-mean-square deviation (RMSD) between CDR-H3 of the parental VHH prediction with the CDR-H3 of the FWR2 variant prediction (Fig. 6b). The Omegafold models of the four VHHs, which did not show large change in Δ*T*_m_, also did not show any large change in CDR-H3 conformation. In conclusion, the Δ*T*_m_ together with the Omegafold models suggests that an IGHV3S53 like FWR2, carrying 37Y and 47L, can have a negative impact on the stability of some VHHs with a kinked CDR-H3 by disturbing the CDR-H3 conformation.

## Discussion

The heavy-chain antibody immune repertoire in camelids is drastically skewed in their VH and JH segment usage. Only a few VH germlines and one JH segment account for the majority of the circulating VHH-based B-cell receptors^6,7^ (Figs. 4d and 5b). Here we show that in addition to the skewed germline usage, a structural polarization exists in the VHH antibody immune repertoire. Using the previously identified τ101 and ɑ101 angles for conventional two-chain antibodies^24^, we were able to classify VHH structures into two clusters with very distinct CDR-H3 conformations. The numerically larger CDR-H3 cluster contains structures with a kinked CDR-H3 loop conformation. The CDR-H3 loops are longer and fold over, forming conserved interactions with FWR2 (Figs. 1c and  2d), which have been recognized previously as a typical CDR-H3 conformation in VHH antibodies^18–20^. The second cluster contains VHH structures with an extended CDR-H3 conformation which exhibits little interaction with the framework. Extended CDR-H3 loops are on average shorter than the CDR-H3 loops of kinked conformations. The CDR-H3 loop conformation impacts antigen binding. VHH domains utilize mostly a convex binding mode likely to compensate for the absence of a light chain^17^. To maintain the convex binding mode, VHH/antigen structures with an extended CDR-H3 conformation utilize the solvent-exposed FWR2 for antigen binding, while VHH structures with a kinked CDR-H3 loop are less likely to do so (Fig. 3). The result is that antibodies with an extended CDR-H3 loop utilize a larger paratope area for antigen binding than antibodies with a kinked CDR-H3 (Table 1). Indeed, the difference in antigen binding between VHHs with kinked and extended CDR-H3 loop has been previously noted and used in the design of a ribosome display library to diversify antibody binding modes^32^.

The polarized VH usage in the llama immune repertoire and the polarized structural repertoire are linked and the two distinct structural CDR-H3 conformational clusters show a strong correlation with VH germline usage. Antibodies derived from the predominant IGHV3-3 germline carry almost exclusively a kinked CDR-H3 conformation while antibodies derived from IGHV3S53, another dominant VH germline, have mostly an extended CDR-H3 loop (Fig. 4). Finally, while underrepresented in our structural dataset, VHHs derived from the third dominant germline family represented by IGHV3S61, IGHV3S65 and IGHV3S66, all germlines encoding an additional cysteine in CDR-H2, which often forms disulfide bonds with CDR-H3 (Supplementary Fig. 7a, b), behave similarly as IGHV3-3-derived antibodies and carry also mostly kinked CDR-H3 loops (Supplementary Fig. 7c).

We demonstrate that VDJ recombination biases during B-cell development are unlikely the cause for the correlation between VH germline usage and the CDR-H3 conformation. Both IGHV3-3 and IGHV3S53 segments appear to recombine with similar frequency with different JH segments, predominantly with JH4 (Fig. 5b). We do not observe large differences in the usage of long and short DH germlines between VH3-3 and VH3S53 based antibodies suggesting that a VDJ usage bias where VH3-3 preferably recombines with long DH-segments leading to longer CDR-H3 loops and a kinked H3 conformation does not exist (Fig. 5c). Furthermore, exonuclease activity and random N-nucleotide incorporation alter the junction of the VH, DH, JH segment during VDJ recombination. Exonuclease trimming has been reported to be sequence-dependent and thus might vary between different germlines, while N-nucleotide addition is expected to be random^33,34^. From a detailed analysis of the VDJ junctions of IGHV3-3 and IGHV3S53-derived antibodies that carry J-segments which are all derived from JH4, we observed differences in trimming in the JH4 segment but not in the two VH segments (Fig. 5). If a trimming bias originating from sequence composition differences is the cause of CDR-H3 loop length differences, we would expect the opposite, that the JH4 segment trimming is the same and the VH segment differs. We, therefore, conclude the observed differences in the VDJ junction between IGHV3-3 and IGHV3S53-derived antibodies—differences in the number of random N-nucleotide insertions and differences in JH4 trimming—are not a product of VDJ recombination biases.

If VDJ recombination biases are unlikely to explain the correlation between CDR-H3 conformation and germline usage, selection pressures that occur during B-cell development might provide an explanation. The ontogeny of domain antibodies in camelids is not well understood, but VHH antibodies likely share their development with regular VH/VL antibodies in llama^35^, and thus similar selection pressures shape the VHH immune repertoire during B-cell development. One major selection step is the removal of autoreactive or multi-specific B-cells^36,29^ but also the interaction with chaperones and chaperone-like molecules such as the surrogate light chain during early B-cell development shapes the immune repertoire^37^. Finally, antigen-experienced B-cells often undergo affinity maturation which changes the biophysical properties of antibodies^38^.

We propose that selection pressure during B-cell development in conjunction with the biophysical properties of FWR2 shape the VHH CDR-H3 loop conformation. IGHV3-3-derived antibodies, which have a hydrophobic FWR2 might be selected to have longer CDR-H3 loops, which adopt a kinked CDR-H3 conformation covering FWR2. This notion is supported by previous work which established a correlation between CDR-H3 loop length and aggregation propensity in VHH demonstrating that VHHs with longer CDR-H3 loops are less-aggregation prone^39^. In contrast, IGVH3S53-derived antibodies are selected to have shorter CDR-H3 loops with an extended conformation as their hydrophilic FWR2 is less likely to provide the hydrophobic scaffold required for the kinked CDR-H3 conformation. Indeed, a recent structural study demonstrated for one particular VHH that the FWR2 composition impacts the kinked CDR-H3 conformation thereby drastically reducing the *T*_m_ of the VHH^29^. We demonstrated that the FWR2 composition impacts also other VHHs with a kinked CDR-H3 suggesting that a hydrophobic FWR2 as it occurs in IGHV3-3 stabilizes a kinked CDR-H3 in many VHHs (Fig. 6).

In summary, our work shows that the composition of the framework region of VHH antibodies clearly associates with the conformations of the CDR-H3 loop. We speculate that the likely cause is selection pressures during B-cell development. Similar impacts of the framework region of the antibody on CDR-H3 loop conformation might be a common phenomenon in antibody repertoires. For example, VH germline-dependent CDR-H3 length bias has been described in human antibody repertoires^40^ suggesting that a similar impact of the framework on the CDR3 conformation as in camelids could exist.

## Methods

### Structural analysis of VH domains

A complete set of PDB deposited Chothia numbered VHH structures was obtained from the Sabdab database (download date Feb 07, 2022)^41^. PDB file handling, structural alignments, bond angle calculation, and contact map generation were performed in R using the Bio3D package^42^. Using metadata provided by Sabdab, structures labeled as originating from *llama* or *alpaca* were retained. PDB files containing multiple VHH structures were split into multiple single files containing only one VHH structure. This dataset was then used to generate a unique set of structures using a 100% sequence identity as a cut-off. The structures of the unique dataset are derived from 236 studies that were published between 1996 and 2022. IGHV germlines were assigned to structures using IgBlast^43^. Solvent-accessible surface areas were calculated using freeSASA^44^. Secondary structure annotations were assigned using DSSP^45^. Sequence logos were generated using the ggseqlogo package^46^. Data was plotted using ggplot2^47^. Structures were visualized using Pymol (Schrödinger). An overview of the number of structures used in the different analysis steps can be found in Supplementary Fig. 10.

### Alphafold and Omegafold structure prediction

Alphafold version 2.2^28^ was used to predict the structures of VHH sequences. To test Alphafold’s ability to predict CDR-H3 conformation 41 kinked and 47 extended structures were randomly chosen from the unique VHH structure dataset. To avoid biases in the prediction stemming from the use of structural templates, Alphafold was run using the -max_template_date=1900-01-01 option. The highest-ranked prediction was used for further analysis. Chothia numbering, structural alignment, rmsd calculations, and determination of bond angles were performed using R/Bio3d^42^.

For 99% of all structures the RMSD over the whole VHH domain between the experimentally and predicted structure was below 1 Å (Supplementary Fig. 8a). A visual inspection of the CDR-H3 loop conformation of the predicted structures suggests that Alphafold correctly predicted the overall CDR-H3 loop conformation (Supplementary Fig. 8b, c). To determine if the ɑ101 and the τ101 angles could be used to classify the CDR-H3 loop structures into a kinked and extended conformation, we inspected the per residue estimate of confidence (pLDDT) of CDR-H3 of the Alphafold predictions. While regions with a pLDDT >70 are expected to contain correct backbone prediction^28^, we noticed that in some predictions the pLDDT drops below 70 at positions 100×, 101, and 102 (Supplementary Fig. 8d, g). A closer inspection of the ɑ101 and the τ101 angles for the predictions reveals that structures where the pLDDT for at least one of the positions at the stem of CDR-H3 (100x, 101, and 102) drops below <70 had a higher chance to exhibit a wrong τ101 and ɑ101angle (Supplementary Fig. 8e, h) and the predicted CDR-H3 loop had also a higher RMSD when compared to the experimentally determined model (Supplementary Fig. 8f, i). Using a pLDDT cut-off for positions 100x, 101, and 102 in the Alphafold predictions we determined that Alphafolds false negative and false positive rate to classify kinked structures is 6% and 9%, respectively, while it is 8% and 6%, respectively, for extended structures. The analysis is based on 35 kinked and 36 extended structures.

Predictions for sequences obtained from the llama immune repertoires were obtained using the max_template_date=2022-05-31 option to include structural templates in the prediction. Predictions where the pLDDT for any of the residues 100x, 101, and 102 dropped below 70 were excluded from further analysis.

For structure prediction using Omegafold, Omegafold v1.1.0^30^ was used. To calculate the RMSD of CDR-H3, the prediction models were Chothia numbered and the respective wildtype VHH prediction was aligned with the mutant VHH prediction model using R/Bio3D, followed by RMSD calculation using residues 96 to 101.

### Repertoire sequencing library generation from llama blood

Fresh blood samples from two non-immunized llamas were obtained from a commercial source (Lampire biological laboratories, Pipersville, PA USA). Llama peripheral blood mononuclear cells (PBMCs) were isolated using Ficoll-PaqueTM PLUS gradient (GE Healthcare) and RNA was extracted using an RNA extraction kit following the manufacturer’s protocol (Qiagen). Unique molecular identifier (UMI) labeled sequencing libraries were generated using a previously described protocol^48^ . RNA was reverse-transcribed using a SMARTer RACE 5’/3’ Kit (Takara Bio) following the manufacturer’s protocol. The Rapid amplification of cDNA ends (RACE) adapter provided in the kit was replaced with a custom race adapter (AAGCAGdUGGTAdUCAACGCAGAGdUNNNNdUNNNNdUNNNNdUCTTrGrGrGrG), which contains UMIs and deoxyUracil modifications. The obtained cDNA was digested with Uracil DNA glycosylase for 40 min at 37 °C and purified using a MinElute PCR Purification Kit (Qiagen). cDNA, roughly estimated to stem from about 20000 B-cells, was used as input for a PCR amplification using the forward primer M1SS (AAGCAGTGGTATCAACGCA) and a 1:1 mixture of reverse primers specific for the IGHC2b and IGHC2c constant region (ATTGGGCAGCCCTGATTTGGTTGTGGTTTTGGTGTCTTGGGTT, ATTGGGCAGCCCTGATTGGAGCTGGGGTCTTCGCTGTGGTGCG). The PCR product was purified using a PCR purification kit (Qiagen) and amplified in a second PCR using the forward primer M1S with a variable N-nucleotide overhang (N_1-4_ CAGTGGTATCAACGCAGAG) and the reverse primer Z (N_1-4_ ATTGGGCAGCCCTGATT). The PCR product was again PCR purified using a PCR purification kit (Qiagen). Sequencing libraries were generated using the NEBNext Ultra DNA Library Prep Kit (NEB) following the manufacturer’s protocol. The obtained sequence libraries were purified using an  agarose gel and a gel extraction kit (Qiagen). The libraries were pooled and sequenced on an Illumina Miseq sequencer using an asymmetric long read option (400 x 100 bp).

### Repertoire sequencing data analysis

FASTQ files obtained from Miseq sequencing were processed using R/Shortread. First, the UMI for each read was identified. Sequences that carried UMI tags that occurred in less than 9 sequence reads were discarded. Next, consensus sequences were generated from R1 and R2 reads carrying the same UMI tag. Paired consensus R1 and R2 reads were assembled into a single sequence spanning the VHH and part of the hinge region using flash pairwise aligner^49^. IGHV, IGHD, and IGHJ germlines were assigned using IgBlast^43^ and the IMGT-published alpaca reference germline set. Constant domain isotypes were assigned using the following llama hinge-derived sequences and a 100% sequence pattern matching: IGHG1A gaactcaagacaccccaacctcaatcccaa; IGHG1B gaaccacatggaggatgcacgtgtccccaa; IGHG2B gaacccaagacaccaaaaccacaaccacaa; IGHG2C gcgcaccacagcgaagaccccagctccaag. Data was plotted using ggplot2^47^.

### VHH-His expression and purification

All constructs were designed in-house and synthesized by Azenta. The constructs contained the VH domain of the respective VHHs (position 1-113) which were cloned into a pRK vector with a C-terminal 8xHis tag and a CMV promoter using the AgeI and HindIII restriction sites. For expression, Expi293 cells (Thermo Fisher) were transfected transiently with expression vectors according to the manufacturer’s recommendations. The antibody-containing supernatants were harvested 7 days post transfection and purified using Ni-NTA resins.

### VHH-His melting temperature measurement

Melting temperatures were acquired with a Nanotemper Prometheus NT.48 fluorimeter (Nanotemper). Excitation power was pre-adjusted to obtain fluorescence readings above 5000 RFU for fluorescence emission at 330 nm (F330) and 350 nm (F350), and samples were heated from 20 to 95 °C with a rate of 1 °C/min. For all measurements, Prometheus NT.48 series nDSF grade high sensitivity capillaries were used. The sample concentration was adjusted to 0.2 mg/mL. The *T*_m_ values were obtained from Prometheus NT.48 software fits. The protein fluorescence emission ratio of 350 nm/330 nm at the melting curve inflection point was calculated from the first derivative and used as the *T*_m_. Δ*T*_m_ was calculated by *T*_m_(mutant) – *T*_m_(wildtype) formula.

### Statistics and reproducibility

Statistical significances were calculated using the statistical program R and Wilcoxon rank test or Chi-squared test using adjusted *p*-values. If not otherwise noted, error bars represent standard deviation. Significance levels were labeled as follows: n.s non-significant, *<=0.05, **<=0.01, ***<=0.001, ****<=0.0001. Melting temperature of VHHs was verified in 3 technical replicates. Reproducibility was tested using subset analysis. Performing analysis with a subset of structures like VHH structures originating only from llama or alpaca led to similar results. Similarly, for repertoire sequencing, each animal was analyzed separately.

### Reporting summary

Further information on research design is available in the Nature Portfolio Reporting Summary linked to this article.

### Supplementary information

Supplementary Figures

Description of Additional Supplementary Files

Supplementary Data 1

Supplementary Data 2

Reporting Summary

## Data Availability

Numeric source data for the main figures can be found in Supplementary Data 1. Numeric source data for the Supplementary Figures can be found in Supplementary Data 2. PDB IDs of the structures analyzed can be found in Supplementary Data 1. Llama immune repertoire Miseq sequencing data have been deposited to the Sequence Read Archive (SRA) with the accession number PRJNA984324.

## References

[1] Hamers-Casterman C (1993). Naturally occurring antibodies devoid of light chains. Nature.

[2] Muyldermans S (2021). Applications of nanobodies. Annu. Rev. Anim. Biosci..

[3] Trinklein ND (2019). Efficient tumor killing and minimal cytokine release with novel T-cell agonist bispecific antibodies. mAbs.

[4] Austin, R. J. et al. TriTACs, a novel class of T cell-engaging protein constructs designed for the treatment of solid tumors. *Mol. Cancer Ther*. 109–120 (2020). 10.1158/1535-7163.MCT-20-0061 33203731

[5] Koenig P-A (2021). Structure-guided multivalent nanobodies block SARS-CoV-2 infection and suppress mutational escape. Science.

[6] Henry KA (2019). Llama peripheral B-cell populations producing conventional and heavy chain-only IgG subtypes are phenotypically indistinguishable but immunogenetically distinct. Immunogenetics.

[7] Tu Z (2020). Landscape of variable domain of heavy-chain-only antibody repertoire from alpaca. Immunology.

[8] Li X (2016). Comparative analysis of immune repertoires between Bactrian Camel’s conventional and heavy-chain antibodies. PLoS ONE.

[9] Spinelli S (1996). The crystal structure of a llama heavy chain variable domain. Nat. Struct. Biol..

[10] Desmyter A (1996). Crystal structure of a camel single-domain VH antibody fragment in complex with lysozyme. Nat. Struct. Biol..

[11] Conrath KE, Wernery U, Muyldermans S, Nguyen VK (2003). Emergence and evolution of functional heavy-chain antibodies in Camelidae. Dev. Comp. Immunol..

[12] Achour I (2008). Tetrameric and homodimeric camelid IgGs originate from the same IgH locus. J. Immunol..

[13] Deschacht N (2010). A novel promiscuous class of camelid single-domain antibody contributes to the antigen-binding repertoire. J. Immunol..

[14] Govaert J (2012). Dual beneficial effect of interloop disulfide bond for single domain antibody fragments. J. Biol. Chem..

[15] Mendoza MN, Jian M, King MT, Brooks CL (2020). Role of a noncanonical disulfide bond in the stability, affinity, and flexibility of a VHH specific for the Listeria virulence factor InlB. Protein Sci. Publ. Protein Soc..

[16] Mitchell LS, Colwell LJ (2018). Comparative analysis of nanobody sequence and structure data. Proteins Struct. Funct. Bioinforma..

[17] De Genst E (2006). Molecular basis for the preferential cleft recognition by dromedary heavy-chain antibodies. Proc. Natl Acad. Sci. USA.

[18] Sang, Z., Xiang, Y., Bahar, I. & Shi, Y. Llamanade: An open-source computational pipeline for robust nanobody humanization. *Structure* (2021). 10.1016/j.str.2021.11.006 PMC11698024 34895471

[19] Sircar A, Sanni KA, Shi J, Gray JJ (2011). Analysis and modeling of the variable region of camelid single-domain antibodies. J. Immunol..

[20] Bond CJ, Marsters JC, Sidhu SS (2003). Contributions of CDR3 to VHH domain stability and the design of monobody scaffolds for naive antibody libraries. J. Mol. Biol..

[21] Beirnaert E (2017). Bivalent llama single-domain antibody fragments against tumor necrosis factor have picomolar potencies due to intramolecular interactions. Front. Immunol..

[22] Kirchhofer A (2010). Modulation of protein properties in living cells using nanobodies. Nat. Struct. Mol. Biol..

[23] Lameris R (2020). A single-domain bispecific antibody targeting CD1d and the NKT T-cell receptor induces a potent antitumor response. Nat. Cancer.

[24] Weitzner BD, Dunbrack RL, Gray JJ (2015). The origin of CDR H3 structural diversity. Structure.

[25] Mitchell LS, Colwell LJ (2018). Analysis of nanobody paratopes reveals greater diversity than classical antibodies. Protein Eng. Des. Sel..

[26] Chaikuad A (2014). Structure of cyclin G-associated kinase (GAK) trapped in different conformations using nanobodies. Biochem. J..

[27] Morea V, Tramontano A, Rustici M, Chothia C, Lesk AM (1998). Conformations of the third hypervariable region in the VH domain of immunoglobulins. J. Mol. Biol..

[28] Jumper, J. et al. Highly accurate protein structure prediction with AlphaFold. *Nature* **596**, 583–589 (2021). 10.1038/s41586-021-03819-2 PMC8371605 34265844

[29] Kinoshita S (2022). Molecular basis for thermal stability and affinity in a VHH: Contribution of the framework region and its influence in the conformation of the CDR3. Protein Sci..

[30] Wu, R. et al. High-resolution de novo structure prediction from primary sequence. Preprint at 10.1101/2022.07.21.500999 (2022).

[31] Valdés-Tresanco MS, Valdés-Tresanco ME, Jiménez-Gutiérrez DE, Moreno E (2023). Structural modeling of nanobodies: a benchmark of state-of-the-art artificial intelligence programs. Molecules.

[32] Zimmermann I (2018). Synthetic single domain antibodies for the conformational trapping of membrane proteins. eLife.

[33] Jackson KJ, Gaeta B, Sewell W, Collins AM (2004). Exonuclease activity and P nucleotide addition in the generation of the expressed immunoglobulin repertoire. BMC Immunol..

[34] Ralph DK, Iv FAM (2016). Consistency of VDJ rearrangement and substitution parameters enables accurate B cell receptor sequence annotation. PLOS Comput. Biol..

[35] Muyldermans S (2013). Nanobodies: natural single-domain antibodies. Annu. Rev. Biochem..

[36] Nemazee D (2017). Mechanisms of central tolerance for B cells. Nat. Rev. Immunol..

[37] Khass M (2016). VpreB serves as an invariant surrogate antigen for selecting immunoglobulin antigen-binding sites. Sci. Immunol..

[38] Shehata L (2019). Affinity maturation enhances antibody specificity but compromises conformational stability. Cell Rep..

[39] Kunz P (2018). The structural basis of nanobody unfolding reversibility and thermoresistance. Sci. Rep..

[40] Sankar K, Hoi KH, Hötzel I (2020). Dynamics of heavy chain junctional length biases in antibody repertoires. Commun. Biol..

[41] Dunbar J (2014). SAbDab: the structural antibody database. Nucleic Acids Res..

[42] Grant BJ, Rodrigues APC, ElSawy KM, McCammon JA, Caves LSD (2006). Bio3d: an R package for the comparative analysis of protein structures. Bioinforma. Oxf. Engl..

[43] Ye J, Ma N, Madden TL, Ostell JM (2013). IgBLAST: an immunoglobulin variable domain sequence analysis tool. Nucleic Acids Res..

[44] Mitternacht, S. FreeSASA: an open source C library for solvent accessible surface area calculations. *F1000Res* **18**, 189 (2016). 10.12688/f1000research.7931.1 PMC4776673 26973785

[45] Touw WG (2015). A series of PDB-related databanks for everyday needs. Nucleic Acids Res..

[46] Wagih O (2017). ggseqlogo: a versatile R package for drawing sequence logos. Bioinformatics.

[47] Wickham H (2016). ggplot2: Elegant Graphics for Data Analysis.

[48] Turchaninova MA (2016). High-quality full-length immunoglobulin profiling with unique molecular barcoding. Nat. Protoc..

[49] Magoč T, Salzberg SL (2011). FLASH: fast length adjustment of short reads to improve genome assemblies. Bioinforma. Oxf. Engl..

