# Supplementary material for: VHH CDR-H3 conformation is determined by VH germline usage
Source: Commun Biol. 2023 Aug 19;6:864. doi: 10.1038/s42003-023-05241-y (PMC10439903; doi:10.1038/s42003-023-05241-y)
Supplement: Supplementary file 1 — Supplementary Figures [file 42003_2023_5241_MOESM1_ESM.pdf]

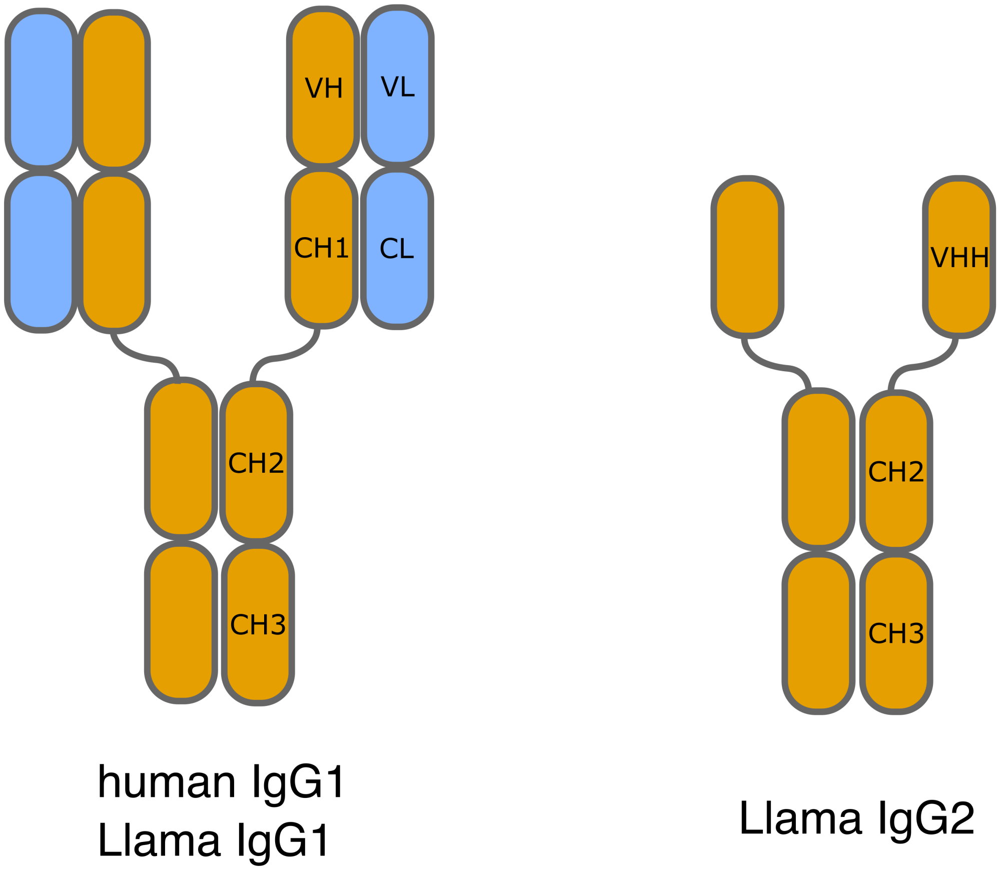

Supplementary Figure 1 Cartoon representations of the domain structure of canonical IgG antibodies represented by human and llama IgG1 antibodies (left) and the domain organization in heavy chain antibodies as found in llama IgG2 antibodies (right).

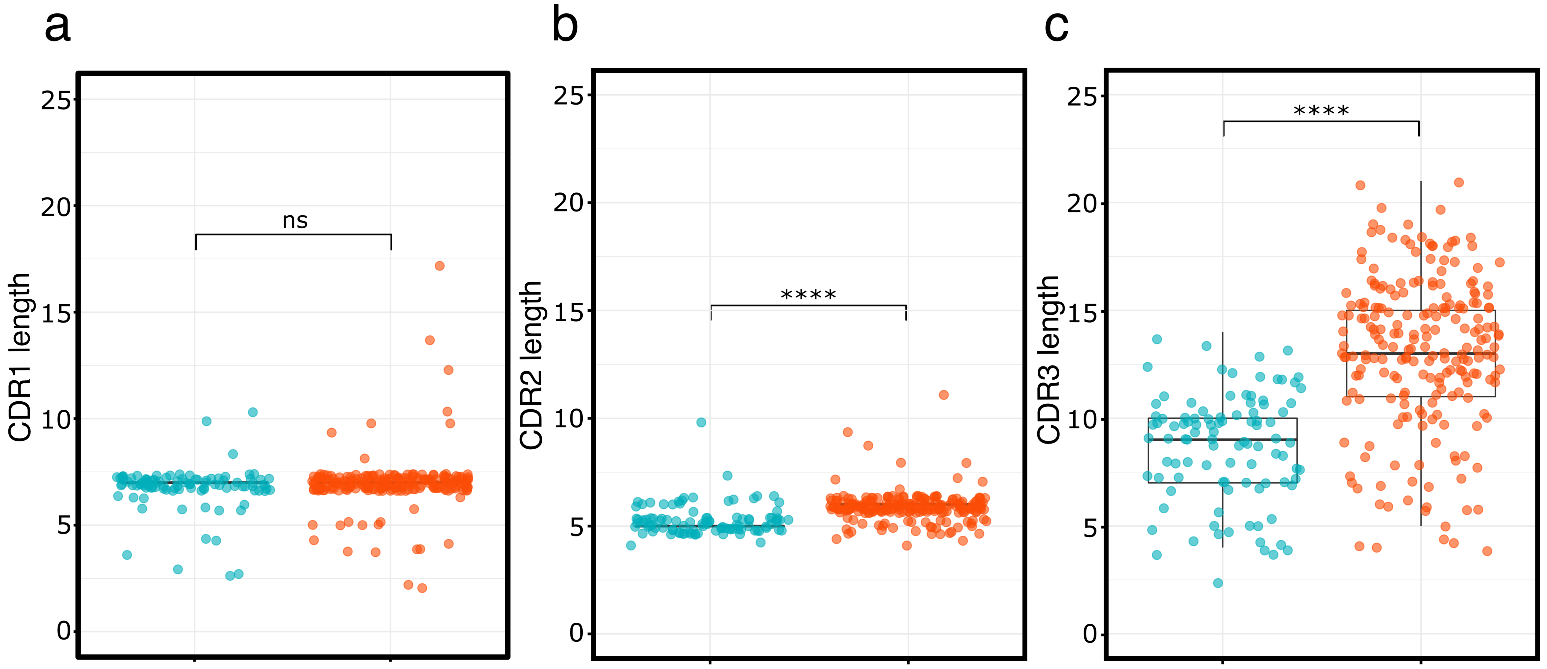

Supplementary Figure 2 a CDR-H1, **b** CDR-H2 and **c** CDR-H3 length distributions of unique llama VHH structure which were classified as extended (teal, n=100) or kinked (red, n=215). CDR definition is based on Chothia numbering scheme (Wilcoxon rank test adjusted p-values, CDR1: 0.057, CDR2: 8.1e-24, CDR-H3: 3.6e-20).

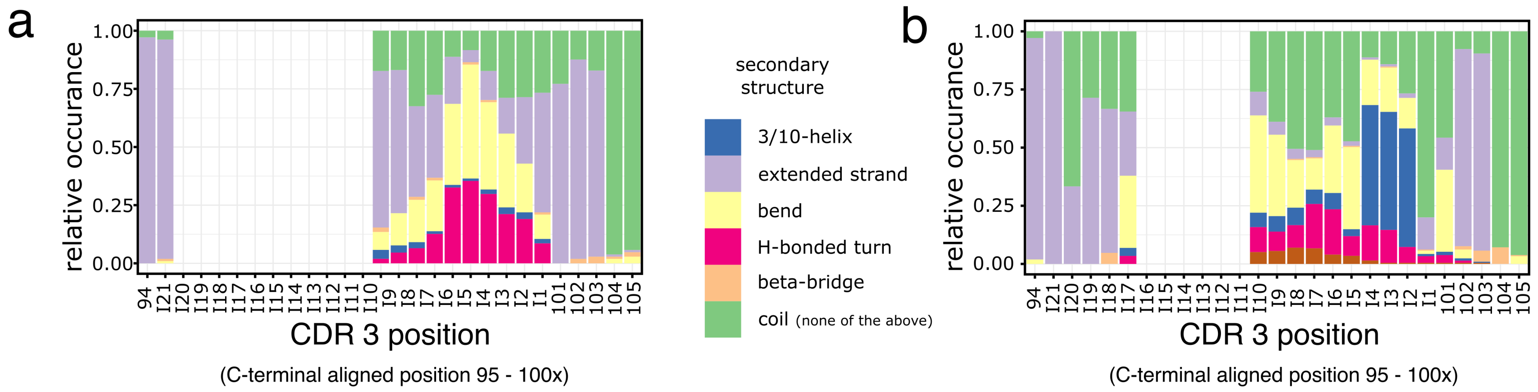

Supplementary Figure 3 a Secondary structure distribution of CDR-H3 position in a unique set of llama and alpaca VHH structures with extended CDR-H3 conformation. Chothia numbering for position 91 – 94 and 101 – 105. The positions in between are aligned from the C-terminus and labeled as I-1 to I-21. **b** As in **a** data shown is for unique llama and alpaca structures with a kinked CDR-H3 conformation.

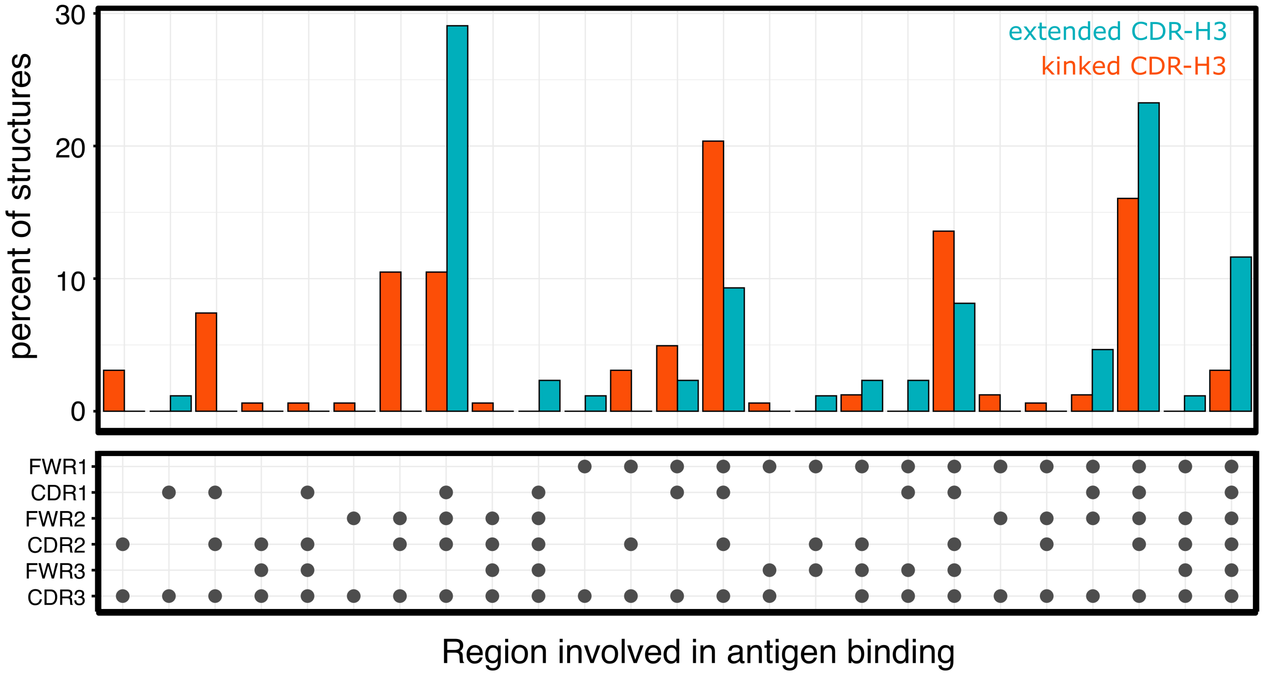

Supplementary Figure 4 Classification of kinked (red) and extended (teal) CDR-H3 VHH structures based on which region of the VHH contributes to antigen binding. The bar graph (upper panel) represents the fraction of structures which have a particular paratope composition. The paratope compositions are shown in the dot plot in the lower panel. Dots represent particular regions which make up a given paratope and are involved in antigen binding.

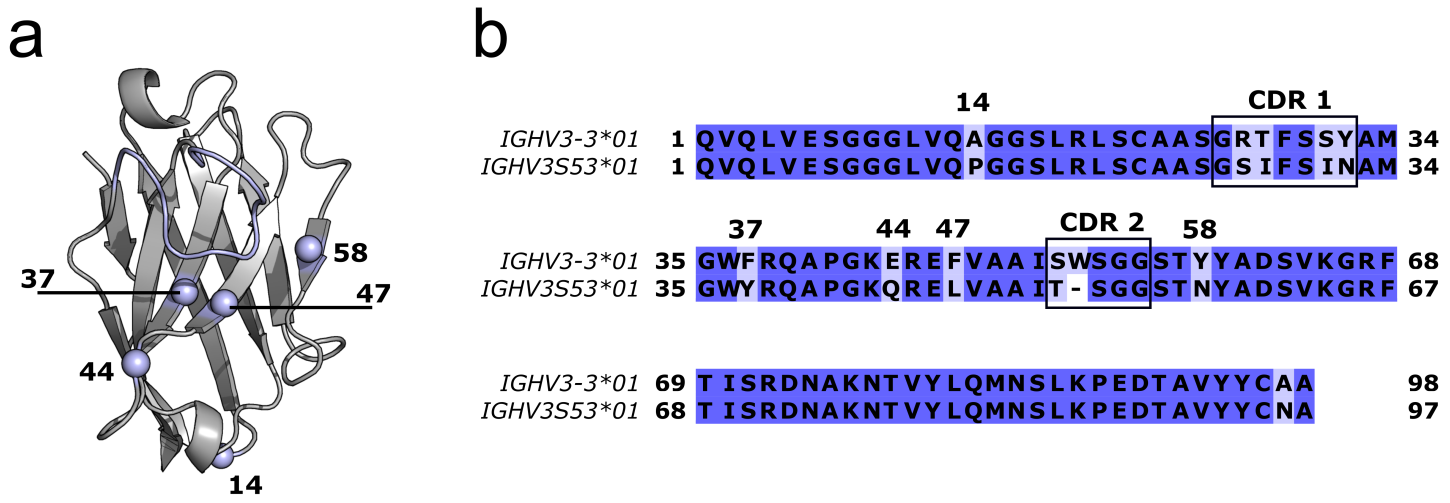

Supplementary Figure 5 a A representative VHH structure with framework positions highlighted which differ between IGHV3-3 and IGHV3S53 (PDB 3K7U.A). **b** Alignment of the alpaca IGHV3-3 and IGHV3S53 germlines. Conserved residues are colored in dark blue, residues which are not conserved in light blue.

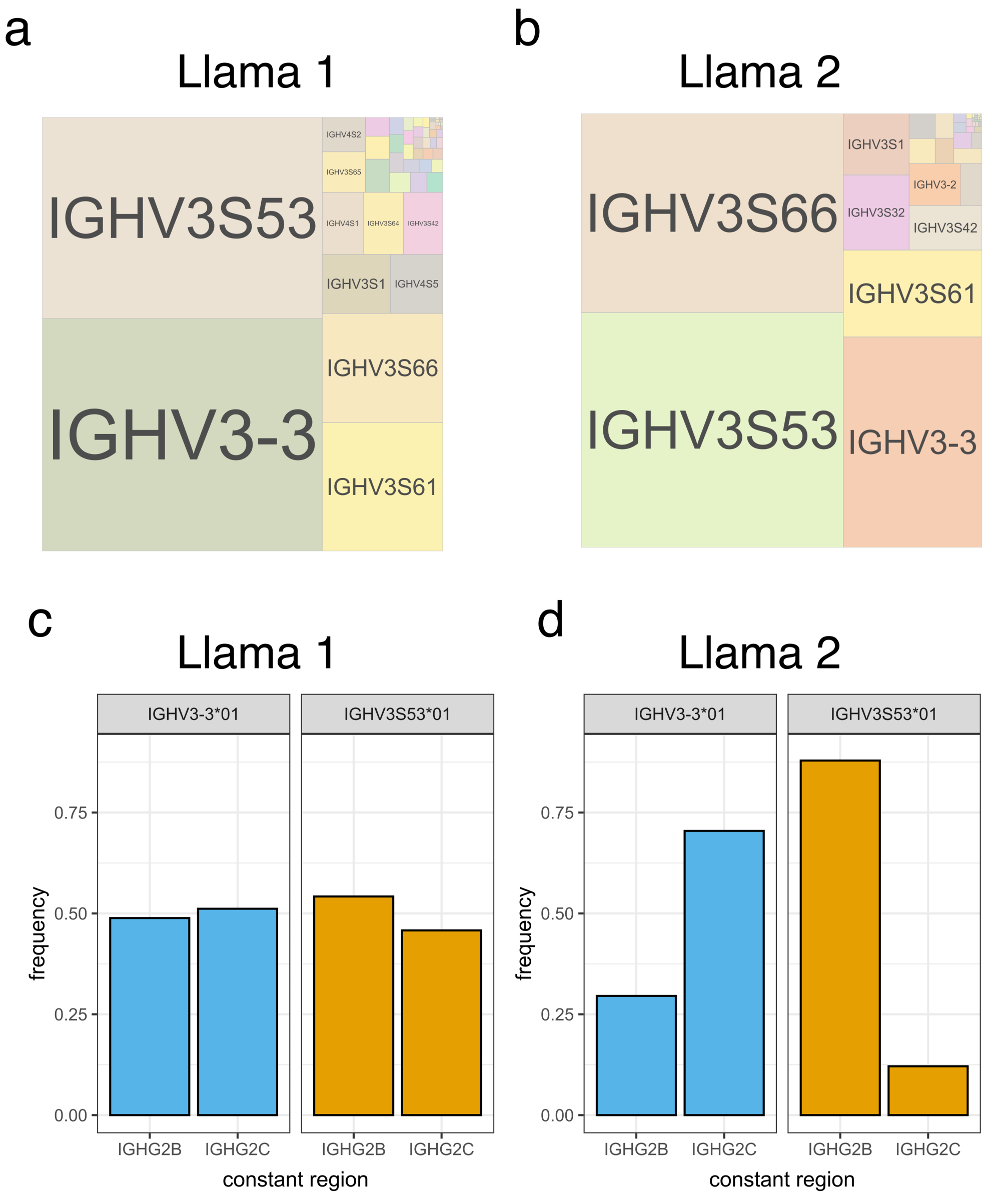

Supplementary Figure 6 a,b Treeplot showing the VH germline usage of the sequenced VHH immune repertoire in the two sequenced llamas. **c, d** Llama IgG2 distribution in the repertoire of llama 1 and 2 for antibodies either derived from IGHV3-3 (blue) or IGHV3S53 (orange) germlines.

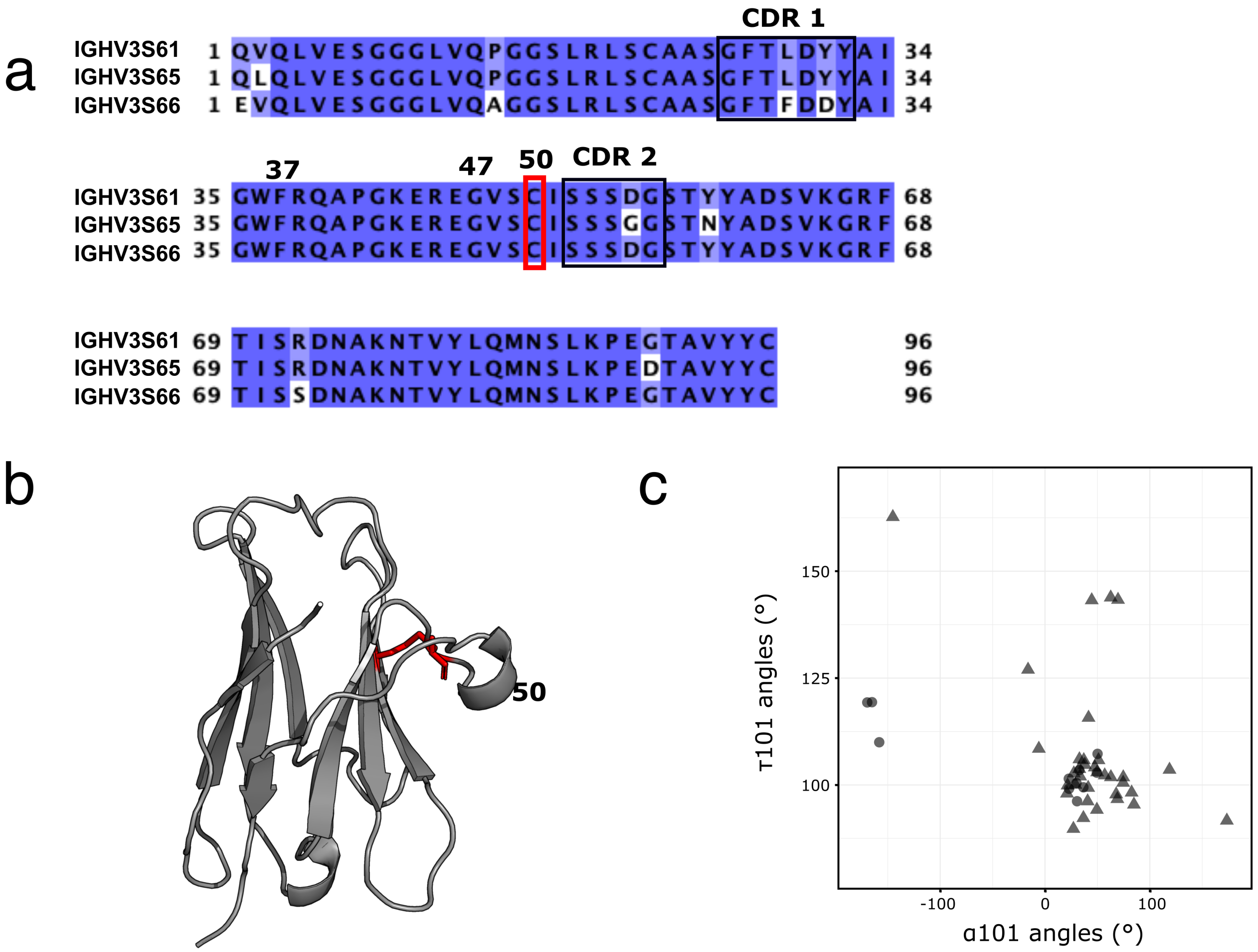

Supplementary Figure 7 a Alignment of alpaca VH germline sequences IGHV3S61 and IGHV3S66. The alignment is colored by sequence conservation; conserved amino acids are colored blue. CDR1 and CDR2 regions according to Chothia definition are boxed in black, the additional cysteine is shown in red. **b** A representative VHH crystal structure (PDB code 7A4D.H) derived from IGHV3S61 with a kinked CDR-H3 and an additional disulfide bond between CDR-H2 and CDR-H3. **c** τ101 and α101 angles for 45 VHH structures derived from IGHV3S61, IGHV3S65 and IGHV3S66 germlines. Structures which have a disulfide bond betweenCDR-H2 and CDR-H3 are symbolized by a ▲, while structures which have an unpaired cysteine in CDR-H2 or which don’t have any additional cysteine are represented by a ●.

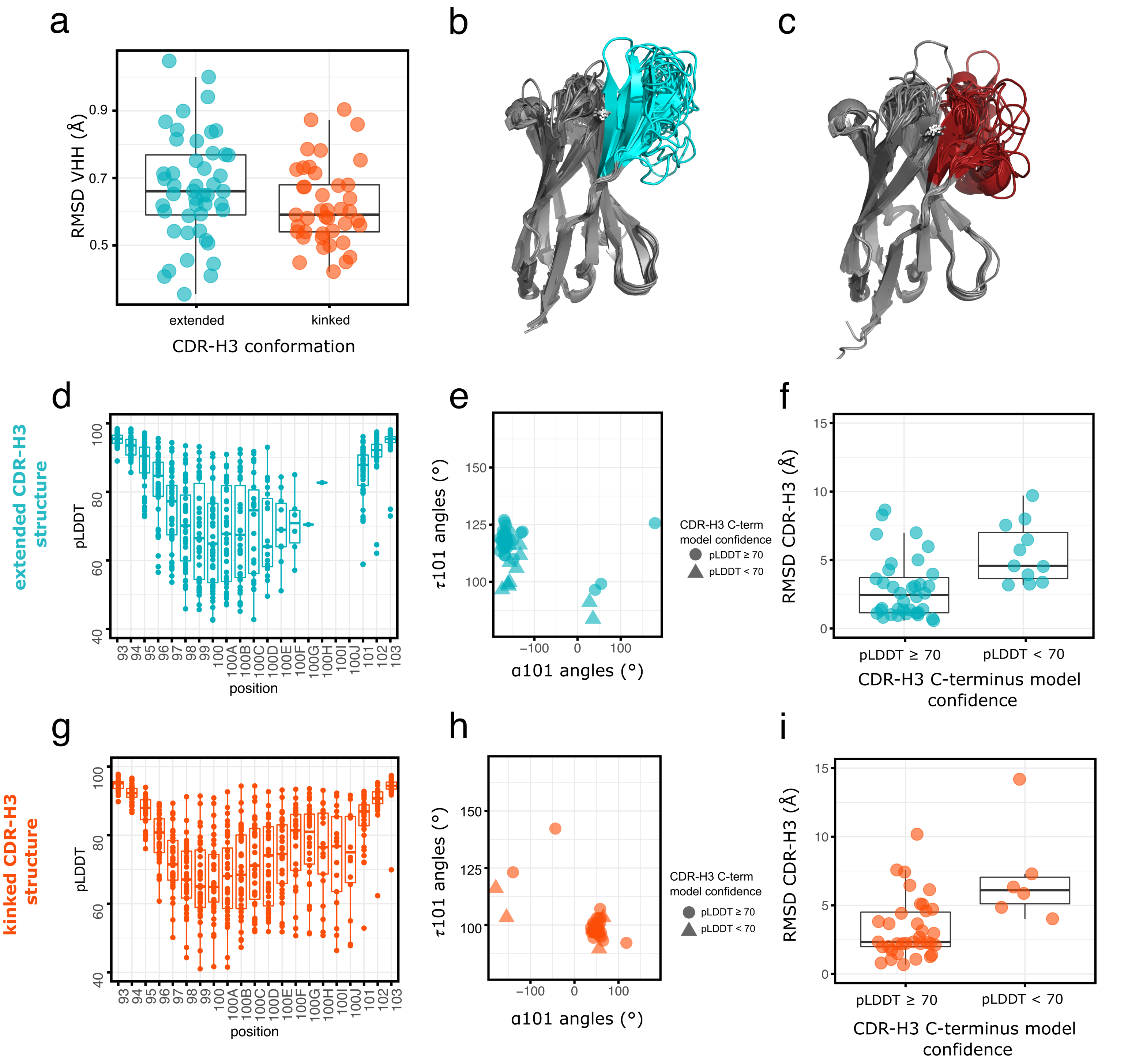

Supplementary Figure 8 a RMSD between experimentally determined VHH structure and the respective predicted model (extended CDR-H3 structures: n=47, kinked CDR-H3 structures: n=40). **b** Structural alignment of all predicted VHH structures which were expected to carry an extended CDR-H3 conformation. The CDR-H3 positions 93 – 102 are highlighted in teal. **c** Structural alignment of all predicted VHH structures which were expected to carry a kinked CDR-H3 conformation. The CDR-H3 positions 93 – 102 are highlighted in red. **d** Per residue estimate of confidence scores for positions 93 – 103 covering CDR-H3 for all predicted structures which were expected to carry an extended CDR-H3 conformation (n=47). **e** α101 and τ101 angle distribution for all predicted structures which were expected to carry an extended CDR-H3 conformation. Predicted structures which had a pLDDT score < 70 at either position 100x, 101 or 102 are labeled with a triangle. **f** RMSD between experimentally determined CDR-H3 structure and the respective predicted CDR-H3 structure for structures (n=47) which had either a pLDDT score below 70 or equal and above 70 at either position 100x, 101 or 102. The RMSD calculation is based on the structural alignment of the whole VHH domain. **g** Per residue estimate of confidence scores for positions 93 – 103 covering CDR-H3 for all predicted structures which were expected to carry an extended CDR-H3 conformation (n=40). **h** α101 and τ101 angle distribution for all predicted structures which were expected to carry an extended CDR-H3 conformation. Predicted structures which had a pLDDT score < 70 at either position 100x, 101 or 102 are labeled with a triangle. **i** RMSD between experimentally determined CDR-H3 structure and the respective predicted CDR-H3 structure for structures (n=40) which had either a pLDDT score below 70 or equal and above 70 at either position 100x, 101 or 102. The RMSD calculation is based on the structural alignment of the whole VHH domain.

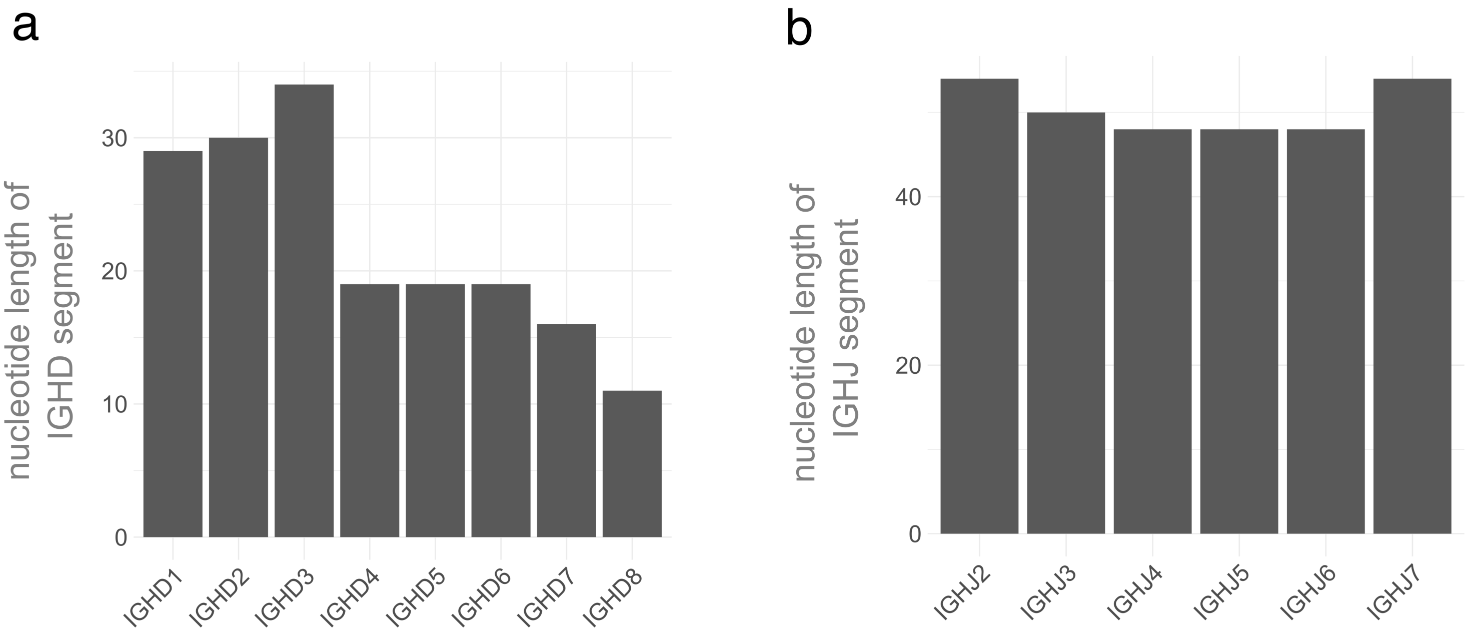

Supplementary Figure 9 a Nucleotide length of alpaca IGHD segments. **b** Nucleotide length of alpaca IGHJ segments.

CDR-H3 conformation analysis

unique PDB structures	385

	kinked	extended	other
classification based on angles	224	110	51
manually removed	9	10	-

Paratope analysis

	kinked	extended
Antigen bound structures	162	86

Germline analysis

	VH3-3	VH3S53
structures derived from germline	175	106
kinked	145	14
extended	14	80
other	16	12

Supplementary Figure 10 Overview of the number of structures used in the analysis workflow starting with the classification of all unique structures in structures with kinked, extended, and other CDR-H3 loop conformation. A small number of structures was removed after manually inspecting the kinked and the extended CDR-H3 clusters as they had very short CDR-H3 or were misclassified. The resulting dataset was used for the subsequent germline and paratope analysis.
