# Supplementary material for: VHH CDR-H3 conformation is determined by VH germline usage
Source: Commun Biol. 2023 Aug 19;6:864. doi: 10.1038/s42003-023-05241-y (PMC10439903; doi:10.1038/s42003-023-05241-y)
Supplement: Supplementary file 2 — Description of Additional Supplementary Files [file 42003_2023_5241_MOESM2_ESM.pdf]

Description of Additional Supplementary Files

File name: Supplementary Data 1
Description: The numeric data used to plot Figure 1-6 and PDB IDs used in the manuscript analysis.

File name: Supplementary Data 2
Description: The numeric data used to plot Supplementary Figure 1-10.
